# Elongation Factor TFIIS Prevents Transcription Stress and R-Loop Accumulation to Maintain Genome Stability

**DOI:** 10.1016/j.molcel.2019.07.037

**Published:** 2019-10-03

**Authors:** Diana Zatreanu, Zhong Han, Richard Mitter, Emanuela Tumini, Hannah Williams, Lea Gregersen, A. Barbara Dirac-Svejstrup, Stefania Roma, Aengus Stewart, Andres Aguilera, Jesper Q. Svejstrup

**Affiliations:** 1 Mechanisms of Transcription Laboratory, The Francis Crick Institute, 1 Midland Road, London NW1 1AT, UK; 2 Bioinformatics and Biostatistics Laboratory, The Francis Crick Institute, 1 Midland Road, London NW1 1AT, UK; 3 Centro Andaluz de Biología Molecular y Medicina Regenerativa-CABIMER, Consejo Superior de Investigaciones Científicas-Universidad Pablo de Olavide-Universidad de Sevilla, Seville, Spain

**Keywords:** TFIIS, transcript elongation, backtracking, RNA polymerase II, transcript cleavage, transcription-associated genome instability, 53BP1, R-loops, DNA-RNA hybrids

## Abstract

Although correlations between RNA polymerase II (RNAPII) transcription stress, R-loops, and genome instability have been established, the mechanisms underlying these connections remain poorly understood. Here, we used a mutant version of the transcription elongation factor TFIIS (TFIIS_mut_), aiming to specifically induce increased levels of RNAPII pausing, arrest, and/or backtracking in human cells. Indeed, TFIIS_mut_ expression results in slower elongation rates, relative depletion of polymerases from the end of genes, and increased levels of stopped RNAPII; it affects mRNA splicing and termination as well. Remarkably, TFIIS_mut_ expression also dramatically increases R-loops, which may form at the anterior end of backtracked RNAPII and trigger genome instability, including DNA strand breaks. These results shed light on the relationship between transcription stress and R-loops and suggest that different classes of R-loops may exist, potentially with distinct consequences for genome stability.

## Highlights

• Inducing transcription stress via TFIIS_mut_ results in reduced nascent RNA synthesis
• TFIIS_mut_ affects expression of long genes and mRNA splicing
• TFIIS_mut_ expression leads to increased R-loop formation and genomic instability
• R-loops may form both anterior and posterior of RNAPII

## Graphical Abstract

**Figure:**
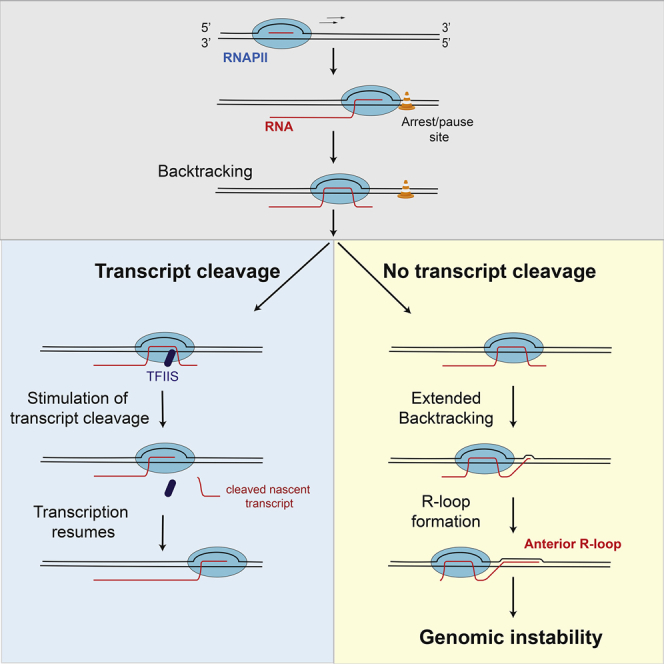

## Teaser

Inducing transcription stress via expression of a TFIIS mutant results in increased R-loop formation. The R-loops may be formed anterior to the backtracking RNA polymerase II and result in increased genomic instability.

## Introduction

In contrast to ATP-driven molecular machines such as helicases, RNA polymerase (RNAP) moves by Brownian motion and may oscillate between productive and backtracked states at various positions on DNA. Transcript elongation is therefore an interrupted process, which includes pausing, backtracking, and arrest (Nudler, 2012) (hereafter often collectively referred to as transcription stress). Although the frequency of such interruption may be low at any individual nucleotide addition site, it must be extremely frequent across the genome. Indeed, deep sequencing of the 3′ ends of nascent RNA isolated with RNAPII elongation complexes (NET-seq) suggested the existence of >2 × 10^5^ detectable pause sites in the compact yeast genome, of which more than 75% were associated with a backtracked polymerase (Churchman and Weissman, 2011). During backtracking, the active site of RNAPII loses control of the RNA 3′ end, which is exuded through a channel below the active site (Kettenberger et al., 2003, Wang et al., 2009).

Backtracked RNAPII is recognized by transcription factor TFIIS (encoded by *DST1* in the yeast *Saccharomyces cerevisiae* and *TCEA1-3* in humans; the functional analogs are GreA and B in bacteria) (Nudler, 2012), which stimulates transcript cleavage by the polymerase active site, thus allowing RNAPII to regain control of the RNA end and resume transcript elongation (Izban and Luse, 1992, Kettenberger et al., 2003, Reines, 1992). Backtracking and transcript cleavage are an integral part of the elongation process, although the likelihood of it occurring is greatly increased by any obstacle to forward translocation, such as nucleotide mis-incorporation, DNA sequences that are difficult to transcribe, nucleosomes, or other DNA-associated factors in the path of RNAPII, including other polymerases (see, for example, Kireeva et al., 2005, Saeki and Svejstrup, 2009, Sigurdsson et al., 2010).

In the absence of transcript cleavage, the ability of backtracked RNAPII to resume transcription is greatly perturbed, which has obvious detrimental effects on transcript elongation itself, but it has also been proposed that backtracked RNAPII is particularly problematic for the maintenance of genome stability (García-Muse and Aguilera, 2016, Nudler, 2012). Most evidence for this idea has been obtained from studies in bacteria or through experiments in eukaryotic cells that only addressed the issue indirectly. For example, Nudler and colleagues provided evidence that genome instability caused by co-directional transcription-replication collision depends on RNAP backtracking in *Escherichia coli* (Dutta et al., 2011). Transcript-cleavage-defective (Gre-deficient) bacterial cells thus have elevated mutation and recombination rates, and their survival depends on the SOS response and error-prone double-strand break (DSB) repair (Dutta et al., 2011, Poteete, 2011). In yeast, the gene encoding TFIIS was uncovered in functional genomics screens for genome instability, with its deletion giving rise to a ∼10-fold increase in gross chromosomal rearrangement (Putnam et al., 2012), through unknown mechanisms.

A study in human cells suggested that *TCEA1* depletion may lead to decreased cell proliferation and apoptosis (Hubbard et al., 2008). Somewhat surprisingly, however, both bacteria and yeast lacking the genes encoding their transcript cleavage factors are viable. In all likelihood, the lack of obvious growth defects indicates an important role for the intrinsic (unstimulated) cleavage activity of the RNAP active site. This idea is supported by work in *S. cerevisiae,* which showed that double mutation of D_290_ and E_291_ (to alanine) in the acidic loop (domain III) of TFIIS (TFIIS_mut_) is lethal and that overexpression of TFIIS_mut_ in a wild-type (WT) background results in a cessation of growth as well (Sigurdsson et al., 2010). Importantly, these mutations not only abrogate normal TFIIS-mediated stimulation of RNAPII-mediated transcript cleavage in the backtracked state but also inhibit the intrinsic cleavage activity of RNAPII (Sigurdsson et al., 2010). More recent enzymatic and biochemical studies showed that TFIIS_mut_ also enhances natural pauses, so that RNAPII spends more time in a backtracked, pre-translocated step during elongation (Imashimizu et al., 2013). Irrespective of the precise underlying mechanism, TFIIS_mut_ thus specifically impedes the rescue of polymerase molecules experiencing transcription stress, “trapping” such RNAPIIs in their backtracked or paused states. It is worth noting that TFIIS also has a role during transcriptional initiation (Guglielmi et al., 2007, Kim et al., 2007, Prather et al., 2005). Importantly, however, this function does not involve transcript cleavage (Guglielmi et al., 2007); TFIIS_mut_ thus only affects transcript elongation, not transcriptional initiation (Sigurdsson et al., 2010).

Given the well-defined and specific effect of TFIIS_mut_ on transcript elongation, we examined the consequences of its expression on a genome-wide scale in human cells. Remarkably, we show that induction of transcription stress via TFIIS_mut_ results in accumulation of RNA-DNA hybrids (R-loops) and increased genomic instability due to such structures.

## Results

### TFIIS_mut_ as a Tool to Study the Effects of Transcription Stress

**Figure 1:**
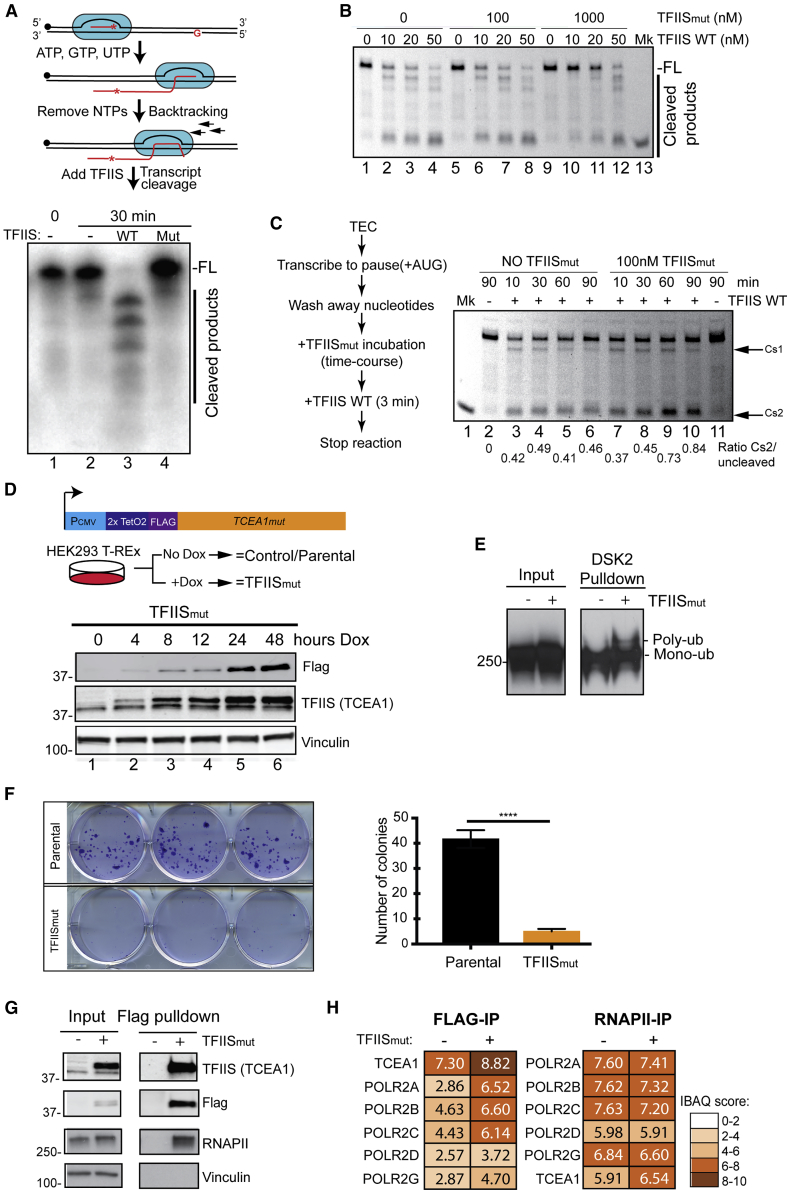
TFIIS_mut_ Interacts with RNAPII and Elicits Transcription Stress (A) Top: schematic of the *in vitro* approach. Bottom: autoradiogram of denaturing PAGE gel of transcript cleavage by different forms of TFIIS. (B) Competition experiment, in which different amounts of TFIIS_mut_ were used to inhibit transcript cleavage induced by WT TFIIS in an 8-min incubation prior to denaturing PAGE analysis. (C) Left: schematic of the approach. Right: incubation of TECs, in the absence of NTPs, with TFIIS_mut_ to test the effect on RNAPII backtracking. After incubation of the TEC with TFIIS_mut_ for the indicated times, the position of RNAPII was determined by inducing transcript cleavage with WT TFIIS for 3 min. Two prominent backtracked positions, Cs1 and Cs2, are indicated by arrows (Cs2 is quantified below). Lane 2 is a 90-min incubation without TFIIS_mut_, and no WT TFIIS, as a control. Similarly, lane 11 is a control incubation with TFIIS_mut_, but again, no WT TFIIS was added. (D) Top: schematics of a cell system to investigate the effect of TFIIS_mut_ expression. Bottom: western blot analysis of doxycycline-induced expression of TFIIS_mut_. The lower band in the TFIIS blot is untagged, endogenous TFIIS. Vinculin is shown as a loading control. (E) Western blot showing mono- and polyubiquitylation of RNAPII (Rpb1 subunit) after enrichment of ubiquitylated proteins by glutathione S-transferase (GST)-DSK2 pull-down. (F) Left: colony-forming ability of TFIIS_mut_-expressing cells, determined by crystal violet staining. Right: quantification of the colony-forming assay (n = 3). Mean ± SEM (bars) values are shown. p values were determined by unpaired t test. (G) Western blot analysis of TFIIS_mut_-FLAG immunoprecipitation. (H) Interaction heatmap, based on intensity based absolute quantification (IBAQ) values, showing TFIIS and RNAPII subunits identified by mass spectrometry after immunoprecipitation of TFIIS_mut_-FLAG (left) or endogenous RNAPII (right) from the TFIIS_mut_-expressing cell line.

Yeast TFIIS_mut_ is incapable of supporting transcript cleavage by RNAPII *in vitro*, and its expression in cells gives rise to transcription stress and a dominant-negative effect on growth (Sigurdsson et al., 2010). To investigate if similar effects are observed in human cells, the point mutations that characterize yeast TFIIS_mut_ were made in human *TCEA1*, generating *TCEA1*_*mut*_ (encoding human TFIIS_mut_). We first analyzed the effect *in vitro* using reconstituted mammalian RNAPII transcription elongation complexes (TECs) and purified, recombinant human TFIIS. Transcript elongation was initially carried out in the absence of cytidine triphosphate (CTP), resulting in RNAPII stopping at the first guanine in the template. After removal of unincorporated nucleotide triphosphates (NTPs), RNAPII was allowed to spontaneously backtrack and transcript cleavage was induced by incubation with TFIIS (Figure 1A, upper). As expected, transcript cleavage by RNAPII was strongly stimulated by WT TFIIS protein, with full-length RNA disappearing and shorter transcript cleavage products appearing (Figure 1A, lane 3), but TFIIS_mut_ failed to stimulate such cleavage (lane 4), indicating that RNAPII cannot be effectively rescued from backtracking in the presence of this form of TFIIS.

To investigate the effect of TFIIS_mut_ on the activity of the WT enzyme, different amounts of TFIIS_mut_ were incubated with nucleotide-depleted, arrested TECs for 10 min, followed by addition of various amounts of WT TFIIS (Figure 1B). Only a large excess of TFIIS_mut_ affected WT-TFIIS-mediated transcript cleavage, suggesting that TFIIS_mut_ is a readily exchanging inhibitor, which does not irreversibly trap RNAPII in an inactive state.

Yeast TFIIS_mut_ inhibits intrinsic RNAPII transcript cleavage (Sigurdsson et al., 2010) and also enhances natural pauses (Imashimizu et al., 2013) so that RNAPII spends more time in a backtracked, pre-translocated step during elongation. To investigate whether this might result in TFIIS_mut_ effectively promoting further backtracking of RNAPII because only forward translocation is impeded, arrested mammalian TECs were incubated with human TFIIS_mut_ over time, before the position of the RNAPII active site was determined by a brief addition of WT TFIIS to induce transcript cleavage (Figure 1C). Characterization by denaturing PAGE showed two primary, backtracked positions (Cs1 and Cs2), and although RNAPII backtracked to a significant extent even in the absence of TFIIS, TFIIS_mut_ clearly enhanced RNAPII backtracking to these positions (Figure 1C, compare Cs1 and Cs2 between lanes 3–6 and 7–10). No intrinsic RNAPII-mediated transcript cleavage was observed even after 90 min in the absence of TFIIS (Figure 1C, lane 2). We were unable to detect the intrinsic transcript cleavage activity of mammalian RNAPII in our experimental system, which precluded a direct investigation of TFIIS_mut_’s inhibitory activity of it. TFIIS_mut_ did not appear to significantly affect pyrophosphate-induced transcript cleavage (Figure S1), which is arguably unsurprising, given that it is a poor inhibitor even of WT-TFIIS-induced transcript cleavage.

### TFIIS_mut_ Expression in Human Cells

In order to establish a system for cellular expression of TFIIS_mut_, we generated a human cell line in which *TCEA1*_*mut*_ was placed under the control of a doxycycline-inducible promoter, allowing its moderate overexpression (Figure 1D). Such expression resulted in RNAPII poly-ubiquitylation (Figure 1E), reflecting the expected increase in transcription stress and likely degradation of RNAPII in a subset of persistently backtracked TECs.

Given the relatively modest level of TFIIS_mut_ expression (Figure 1D), and given that TFIIS_mut_ is a readily exchanging inhibitor (Figure 1B), the effects on cell viability might be expected to be limited. However, we noticed that upon TFIIS_mut_ expression, growth rates declined with time, especially under conditions of low cell density. In order to better assess this effect, we performed colony formation assays. After 11 days of TFIIS_mut_ overexpression, only a few colonies were observed (Figure 1F). This shows that, similar to yeast (Sigurdsson et al., 2010), the TFIIS_mut_ protein has a negative effect on cell growth and viability. For subsequent experiments, induction of TFIIS_mut_ was carried out for 48 h before experimental analysis; at this point, cells grew normally and showed no outward signs of distress.

We also wanted to ensure that TFIIS_mut_ does not have a fundamentally altered interaction with RNAPII. We therefore performed immunoprecipitation of either FLAG-tagged TFIIS_mut_ or endogenous RNAPII from the solubilized chromatin fraction of a nuclear extract. Western blot analysis of the TFIIS_mut_ immunoprecipitates confirmed the specificity of the interaction (Figure 1G), and the protein samples were subjected to mass spectrometry analysis (Table S1). This revealed that the interaction between TFIIS_mut_ and RNAPII is not significantly altered, and only a marginal increase in the overall level of interaction with RNAPII was observed in chromatin upon TFIIS_mut_ overexpression (Figure 1H). This suggests that TFIIS_mut_ competes with the WT protein for association with “stressed” RNAPII during transcript elongation.

### TFIIS_mut_ Pauses RNAPII in the Gene Body

**Figure 2:**
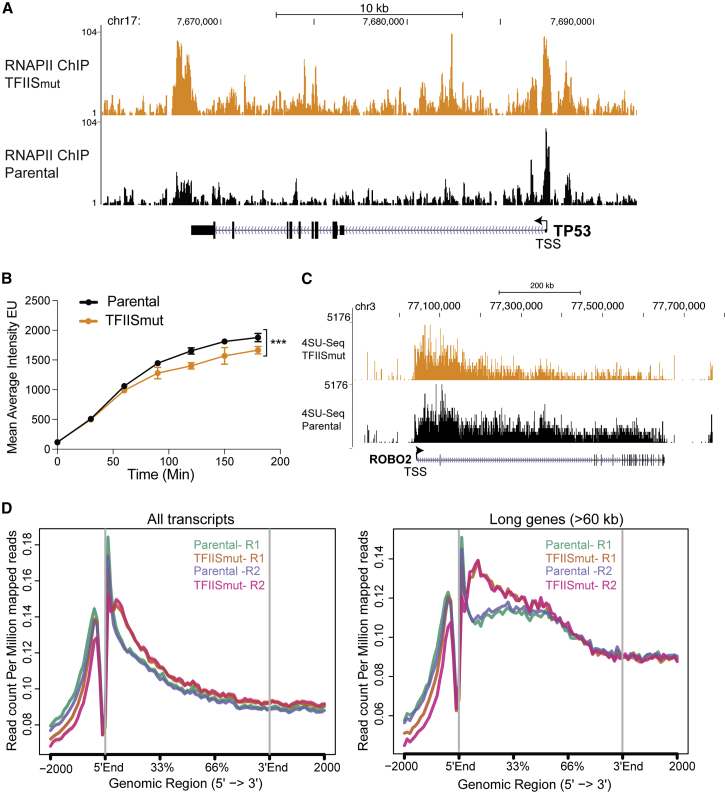
TFIIS_mut_ Pauses RNAPII in the Gene Body (A) Effect of TFIIS_mut_ expression on the RNAPII ChIP-seq profile at the *TP53* gene. TSS, transcription start site. (B) Line plots of mean average 5-EU intensity of nascent RNA labeled for different times with and without TFIIS_mut_ expression. Bars represent ±SEM. p values were determined by two-way ANOVA statistical test. (C) Representative example of the TT-seq profile, across the *ROBO2* gene. Notice the relative accumulation of 5′ end reads in the TFIIS_mut_-expressing cells. Another example is shown in Figure S2B. (D) Metagene TT-seq profile of all transcripts (left) and of long transcripts (>60 kb, right) anchored by 5′ end (0%) and 3′ end (100%) of genes of TT-seq for two biological replicates.

TFIIS dynamically associates with paused or backtracked RNAPII. As suggested by the *in vitro* data of Figures 1A–1C, expression of TFIIS_mut_ would thus be expected to trap RNAPIIs experiencing transcription stress, prolonging pausing and lengthening backtracks stochastically across genes. To investigate the consequences of such events inside cells, we first performed RNAPII chromatin immunoprecipitation sequencing (ChIP-seq) analysis. Upon TFIIS_mut_ overexpression, numerous genes, such as *TP53*, showed increased RNAPII occupancy in the coding region, suggesting problems with transcript elongation (Figure 2A). No obvious features, such as gene length or DNA sequence motifs, were in common among the most affected genes. We also used 5-ethynyl uridine (5-EU) incorporation to measure nascent RNA synthesis. Time-course analysis revealed that in TFIIS_mut_-expressing cells, the overall rate of RNA synthesis is reduced (Figures 2B and S2A).

To further investigate the effect of TFIIS_mut_ on transcription genome-wide, we employed a modified version of 4-thiouridine sequencing (TT-seq) (Schwalb et al., 2016), which allows measurements of nascent RNA synthesis at high genomic resolution. Cells were metabolically labeled with 4-thiouridine (4SU) for 20 min, allowing the subsequent separation of recently transcribed RNA from the overall RNA population, before RNA fragmentation, library production, and deep sequencing (Gregersen et al., 2019) (see STAR Methods for details). The results from the *ROBO2* gene is shown as an example (Figure 2C; see also Figure S2B); RNAPII activity was fairly equally distributed across the gene in parental cells but was relatively depleted toward the 3′ end in cells expressing TFIIS_mut_. Meta-gene profiles revealed that this was a general effect, as indicated by the concentration of RNAPII activity in the first third of genes (Figure 2D, left). Not surprisingly, this was more clearly observed in long genes (Figure 2D, right).

We also used the CDK9- and transcription elongation inhibitor 5,6-dichloro-1-β-D-ribofuranosylbenzimidazole (DRB) in combination with nascent RNA analysis (4SU incorporation), as a variant of DRB/global run-on (GRO) analysis (Saponaro et al., 2014) to analyze RNAPII elongation. Upon release from DRB inhibition, cells were incubated at different time points with 4SU for 10 min (Figure S2C, top). Total 4SU incorporation was decreased at a global level, which was most easily observed 10 min after DRB release (Figure S2C, bottom). Single-gene analysis of RNAPII progression was then carried out for the long *OPA1* gene, with nascent RNA levels measured by qRT-PCR using primers that spanned the length of the gene every ∼5 kb. After 40 min of transcript elongation, a markedly lower level of RNAPII activity was observed toward the end of the gene in TFIIS_mut_-expressing cells (Figure S2D), indicating that RNAPII on average takes longer to run into this area of the gene when transcript cleavage is inhibited by TFIIS_mut_ expression.

Taken together, the results from the different genome-wide and gene-specific analyses described above support the idea that inducing transcription stress via TFIIS_mut_ results in a global reduction of nascent RNA synthesis and slower average elongation across genes, in all likelihood caused by increasing the time spent by RNAPII in pause and backtracking mode.

### Changes in Gene Expression and Splicing in Response to TFIIS_mut_ Expression

mRNA processing is tightly coupled to elongation, with mRNA processing factors likely being deposited by RNAPII so that slow elongation may affect alternative cassette exons splicing and termination (Dujardin et al., 2013). We therefore also investigated the effect that TFIIS_mut_ expression has on mRNA processing by deep sequencing of libraries generated from mRNA.

First, although several genes were differentially expressed in the TFIIS_mut_-expressing cells compared to control, the differences were modest (Figure S3A; Table S2). Tellingly, however, the median length of the 124 upregulated genes was 18.1 kb, while that of the 145 downregulated genes was no less than 193.8 kb (Figure S3B), again underscoring the selective effect of TFIIS_mut_ on transcript elongation.

**Figure 3:**
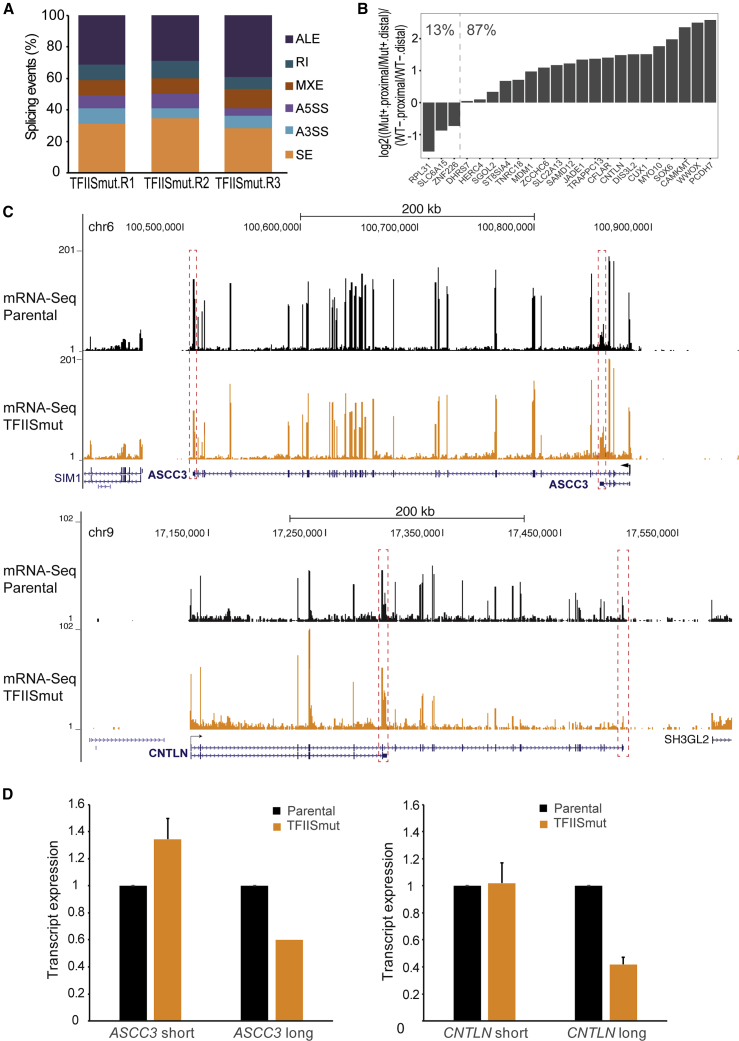
Alternative Last Exon Splicing in TFIIS_mut_ Cells (A) mRNA isoform expression-changes in TFIIS_mut_-expressing cells detected by MISO analysis of mRNA-seq. A3SS, ALE, alternative last exon, alternative 3′ splice site; A5SS, alternative 5′ splice site; MXE, mutually exclusive exons; RI, retained exon; SE, skipped exons. (B) Relative expression differences of terminal exons associated with common ALE events induced by transcript cleavage inhibition. The ratio to distal exon was calculated for the TFIIS_mut_ sample and normalized to that in parental cells. (C) Representative examples of ALE events in RNA-seq profiles (top, *ASCC3*; bottom, *CNTLN*). (D) qRT-PCR validation of isoform expression. GAPDH-normalized and relative to parental conditions (n = 3). Mean ± SEM (bars) values are shown.

Second, in order to quantitatively measure the relative expression of transcript isoforms (mRNA splicing), we used the mixture of isoform (MISO) model (Katz et al., 2010). Relative to parental cells, hundreds of splicing events were affected by TFIIS_mut_ expression (Table S3), but with relatively few changes in cassette exon splicing and with alternative last exon (ALE) events being among the most frequent, accounting for approximately one-third of all splicing events recorded in all three replicates (Figure 3A). ALE transcript isoforms are characterized by differential 3′ terminal exon usage; i.e., they are mechanistically a consequence of alternative transcriptional termination. We analyzed the common ALE events from all three biological replicates and observed a consistent, relative upregulation of shorter transcript isoforms (Figure 3B). The relative expression of the isoforms of two gene examples, *CNTLN*, which was among the genes that showed upregulation of a shorter isoform, and *ASCC3,* which was identified by manual inspection as an additionally affected gene, is shown in Figure 3C; qRT-PCR experiments confirmed the results at those genes (Figure 3D). Interestingly, these results suggested that the relative upregulation of short isoforms may be caused by a relative failure of RNAPII to reach the end of these long genes (i.e., loss of the long isoforms), presumably through a decrease in RNAPII processivity rather than an increase in the short isoforms per se.

We conclude that TFIIS_mut_ expression affects gene expression at long genes and that it, not unexpectedly, also affects mRNA splicing and termination.

### Expression of TFIIS_mut_ Leads to Increased R-Loop Formation

**Figure 4:**
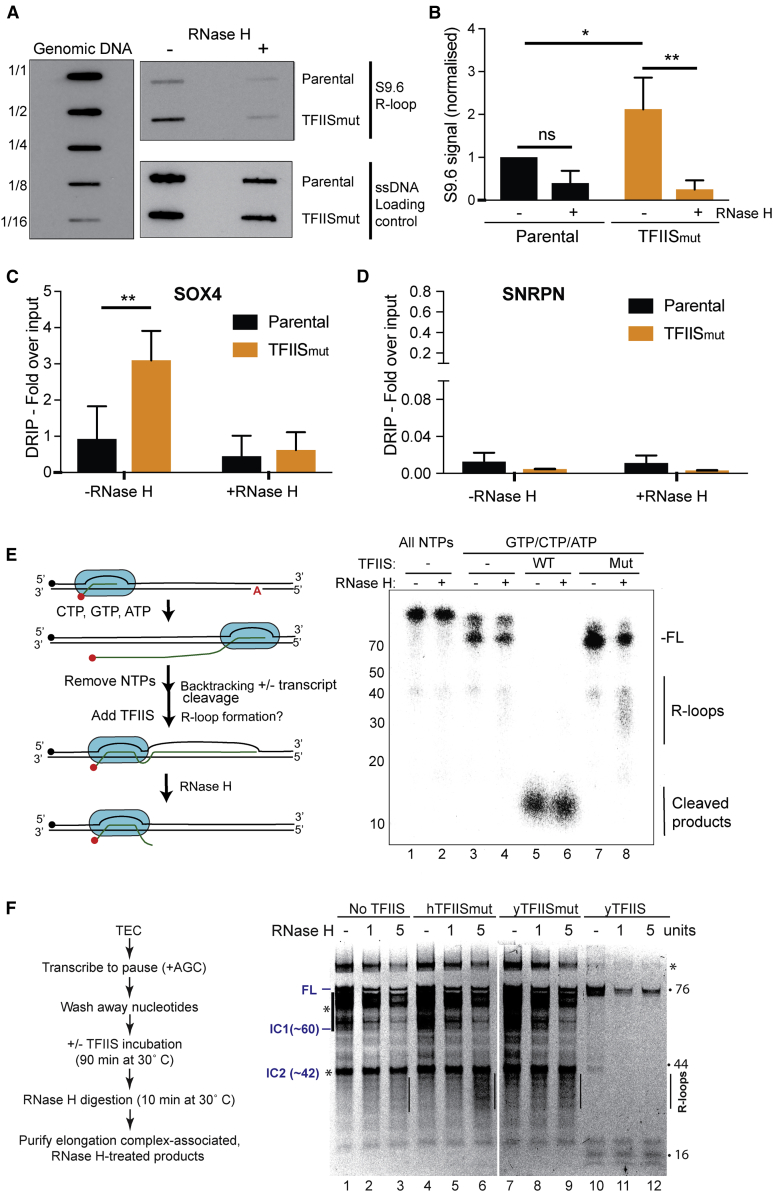
TFIIS_mut_ Expression Results in an Accumulation of R-Loops (A) RNA or DNA hybrid slot-blot of genomic DNA from TFIIS_mut_ and parental cells, ±RNase H. S9.6 antibody was used to detect RNA or DNA hybrids (upper panel on right) with single-strand DNA antibody (bottom panel) as a loading control. Serial dilutions of genomic DNA (1/1 = 4 μg) were probed with S9.6 antibody for standards (left panel). (B) Fold enrichment in RNA or DNA hybrids compared with control (n = 3). Mean ± SEM (bars) values are shown. p values were determined by unpaired t test. (C and D) DRIP-qPCR analysis of R-loop induction at the *SOX4* gene (C) and the *SNRPN* gene (D) (n = 3). Mean ± SEM (bars) values are shown. p values were determined by two-way ANOVA statistical test. (E) Left: schematic of idealized experiment. Radioactive label is denoted by red dot and the biotin tag on DNA with a black dot. The position of the first adenine in the transcript is also indicated. Right: R-loop detection by denaturing PAGE after addition of TFIIS proteins and RNase H to yeast TECs assembled *in vitro*. Ambion RNA size markers are indicated on the left for approximate RNA sizes. Positions of full-length product (FL), R-loops, and cleavage products are indicated on the right. (F) Left: experimental scheme; similar to that of (E) but involving purification via the biotin tag after RNase H digestion. Right: R-loop detection by denaturing PAGE after addition of TFIIS proteins and RNase H to yeast TECs assembled *in vitro*. Approximate RNA sizes RNA and position of R-loops are indicated on the right and next to relevant lanes. Asterisk-bar denotes irrelevant pausing sites of unknown origin, including IC1 and IC2. See Figure S5 for detailed schematic explanations.

Several kinds of transcription perturbation can result in the accumulation of R-loops in eukaryotic cells (Santos-Pereira and Aguilera, 2015), but whether transcriptional pausing and backtracking can induce them has not been directly investigated. We therefore next analyzed if TFIIS_mut_ expression results in the accumulation of such DNA-RNA hybrids. Interestingly, slot blot analysis of isolated genomic DNA using the hybrid-specific S9.6 antibody revealed a clear increase in RNase-H-sensitive R-loops after TFIIS_mut_ expression (Figures 4A and 4B). We also used the S9.6 antibody to detect R-loops by immunostaining of these cells. As expected from the data in Figures 4A and 4B, nucleoplasmic R-loop staining intensity was consistently markedly higher in the TFIIS_mut_-expressing cells (Figures S4A and S4B).

We also used DNA-RNA immunoprecipitation (DRIP) to investigate the accumulation of R-loops at individual genes. We first investigated some of the genes that have previously been shown to accumulate R-loops, such as *APOE*, *RPL13A*, and *BTBD19* (Bhatia et al., 2014, Ginno et al., 2013, Herrera-Moyano et al., 2014) (Figure S4C). A consistent trend in R-loop accumulation at these genes was observed in TFIIS_mut-_expressing cells, and, importantly, RNase H treatment again abolished this accumulation. Utilizing our previously generated RNAPII ChIP-seq data (Figure 2), we also identified a further gene with R-loop accumulation based on two criteria: first, we filtered for genes that had markedly increased RNAPII density in the gene body by ChIP, suggesting many backtracked and/or stalled RNAPIIs, and, second, we identified the subset of those genes that also have many or large CpG islands, as R-loops often form in CG-rich regions (Ginno et al., 2012). The *SOX4* gene convincingly met both criteria (Figure S4D) and, gratifyingly, DRIP-qPCR analysis showed marked R-loop accumulation at this gene as well (Figure 4C). By contrast, the *SNRPN* gene has previously been shown by others to *not* accumulate R-loops (Bhatia et al., 2014, Herrera-Moyano et al., 2014) and was used as a negative control for these results (Figure 4D).

R-loops are generally thought to arise by re-annealing of the nascent RNA transcript with the DNA template *behind* the elongating polymerase. Indeed, disruption of factors that are important for packaging of the nascent RNA into mRNP particles often gives rise to significant R-loop accumulation (Santos-Pereira and Aguilera, 2015). Although an effect on RNA packaging cannot be ruled out, TFIIS_mut_ expression would not be expected to affect such transactions. Instead, it would be predicted to trap backtracked RNAPII and potentially stimulate further backtracking and thus potentially result in the formation of R-loops via nascent RNA that is in front of the polymerase. Alternatively, backtracked RNAPII might be dissociated or degraded, potentially leaving the released, nascent RNA to form R-loops. Directly investigating whether R-loops form anterior or posterior to RNAPII *in vivo* is unfortunately not possible with presently available techniques, so we instead set out to test if an effect of TFIIS_mut_ on R-loop formation could be observed with pure proteins *in vitro*. For this purpose, we used the previously described *in vitro* transcription assay, this time with a template in which RNAPII elongated from a radio- or fluorescent-labeled RNA oligonucleotide up to a position with an adenine in the coding strand where it stopped, because no UTP was included in the reaction (generating a 76-nt transcript) (Figure 4E, left). Because the signal from these reactions was very weak, we used the more active yeast RNAPII for these experiments. After removal of unincorporated NTPs, backtracking (and potentially R-loop formation) was stimulated through the addition of TFIIS_mut_ (and WT TFIIS as control). RNase H was then used to assess if R-loops were formed. Characterization by denaturing PAGE showed that the RNA behind the elongating RNAPII generated few, if any, R-loops under these conditions (Figure 4E, right; compare lanes 1 and 2). Upon removal of nucleotides and the addition of TFIIS_mut_ to allow backtracking, but not transcript cleavage, a weak smear of fragments of a smaller size were visible only in the RNase-H-treated sample, suggesting that R-loops were formed by RNA in front of the backtracking polymerase (Figure 4E, compare lanes 7 and 8). The conclusion that these RNase-H-generated RNA products were anterior R-loops was further strengthened by purification of the TECs after RNase H cleavage, which removes the label from the RNA of posterior R-loops (Figure 4F, compare lanes 6 and 9 with lane 3; see also Figure S5 for detailed description of this experiment). As expected, no anterior R-loops were detected in the samples incubated with cleavage-inducing WT TFIIS, because the transcripts in these conditions are continuously cleaved as the polymerase moves backward (Figure 4E, lanes 5 and 6; and Figure 4F, lanes 11 and 12). Importantly, a small but detectable signal was observed in the samples in which no TFIIS was added (compare Figure 4E, lanes 3 and 4; and Figure 4F, lane 1 versus lane 3), together suggesting that R-loop formation is intrinsic to the backtracking polymerase rather than specific to the conditions where TFIIS_mut_ is present. The fragments produced by RNase H were 30–40 nt, in agreement with the expectations from the RNAPII “footprint”’ (see Figure S5).

Together, these results are consistent with the idea that R-loops accumulate in cells that have defects in transcript cleavage, caused at least partly by retrograde sliding of RNAPII, which allows hybridization of the nascent RNA with the DNA template in front of the polymerase, generating anterior R-loops.

### R-Loops Give Rise to Genome Instability in TFIIS_mut_-Expressing Cells

**Figure 5:**
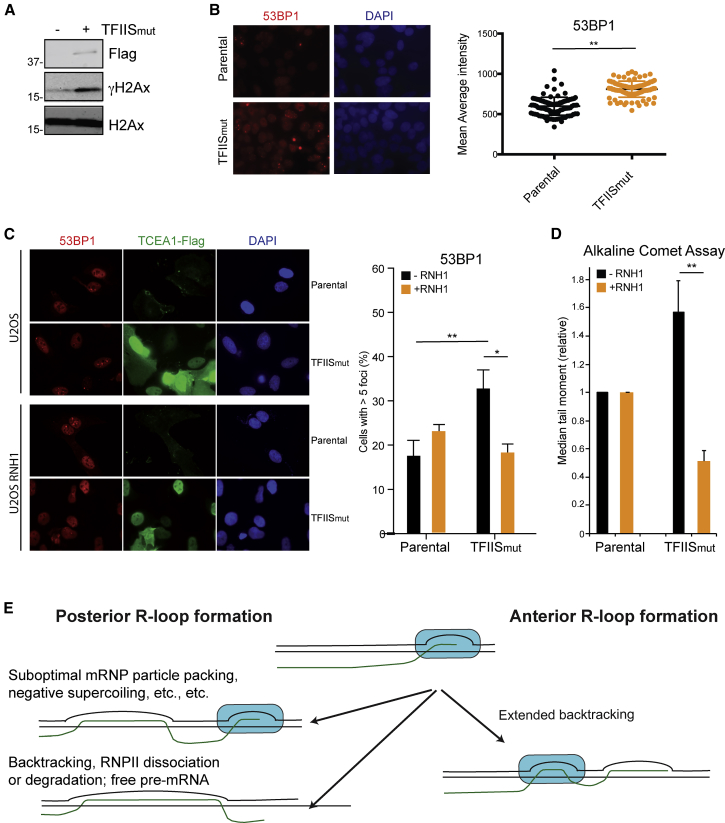
TFIIS_mut_ Induces R-Loop-Dependent DNA Damage (A) Western blot analysis of the chromatin fraction after overexpression of TFIIS_mut_, showing γH2Ax levels. Histone H2Ax is shown as a loading control. (B) Left: immunofluorescence of TFIIS_mut_-expressing cells, stained with antibodies against 53BP1. Right: quantification of average nuclear intensity. p values were determined by unpaired t test. (C) Left: immunofluorescence of U2OS cells transiently transfected with TFIIS_mut_ plasmid, stained with antibodies against 53BP1, ±doxycycline-inducible RNase H1 expression. Right: percentage of cells with more than 5 foci. Mean ± SEM values from at least five independent experiments are shown. p values were determined by unpaired t test. (D) Quantification of DNA breaks after transfection with TFIIS_mut_ assessed by the alkaline comet assay ±RNase H1. Mean ± SEM values from seven independent experiments are shown. p values were determined by unpaired t test. (E) Model for the genesis of different kinds of R-loops in cells.

We finally investigated the impact of TFIIS_mut_ expression and R-loop formation on genome stability. We initially assayed the effect of TFIIS_mut_ expression by detection of phosphorylated H2AX (γH2AX) as a general marker for DNA damage. Upon overexpression of TFIIS_mut_ to induce transcription stress, an increase in chromatin-bound levels of γH2Ax was observed by western blotting (Figure 5A). These results provided the first indications that the increase in pausing and backtracking caused by TFIIS_mut_ expression affects genome stability.

Given these results, we now tested the effect of TFIIS_mut_ expression on the levels of 53BP1, a marker of DNA DSBs (Schultz et al., 2000). TFIIS_mut_-expressing HEK293 cells showed a clear increase in the general staining levels of 53BP1 (Figure 5B). Unfortunately, the HEK293 cells are not suitable for microscopy analysis. For this reason, and also to investigate the phenomenon in an independent cell line, we used U2OS cells for most of the remaining experiments. In these assays, we transiently expressed TFIIS_mut_ and then quantified the accumulation of 53BP1 foci. Gratifyingly, 53BP1-foci-containing cells were clearly increased upon expressing TFIIS_mut_, and this was repressed by concomitant RNase H1 overexpression (Figure 5C), indicating that genome instability was caused by R-loop accumulation. For further evidence of the accumulation of DNA breaks in an R-loop-dependent manner, we directly analyzed the accumulation of DNA strand breaks by single-cell gel electrophoresis (Comet assay) (Collins, 2004). We performed this assay under alkaline conditions to detect both double- and single-stranded DNA breaks. In these assays, expression of TFIIS_mut_ led to a larger tail moment than control cells, and this effect could again be suppressed by overexpression of RNase H1 (Figure 5D), suggesting that transcription stress-induced R-loops result in DNA strand breaks.

We previously used comparative genomic hybridization (CGH) to show that chromosomal rearrangements can occur in response to certain kinds of transcription stress, such as after *RECQL5* or *MLL2* perturbation (Kantidakis et al., 2016, Saponaro et al., 2014). This technique compares different genomic DNA samples for genomic changes such as gains or losses of chromosomal regions or whole chromosomes (Park, 2008). TFIIS_mut_-expressing HEK293 cells were grown in the absence of doxycycline, and the batch was then split in two and grown either in the continued absence of doxycycline or in the presence of it to induce TFIIS_mut_ overexpression. Genomic DNA preparations from the two cell populations were then compared by CGH. Interestingly, we detected no genomic rearrangement upon TFIIS_mut_ expression, either after 48 h or after 7 days of induction.

Altogether, these results suggest that R-loops form and accumulate when transcription elongation is perturbed by inhibiting transcript cleavage, leading to genomic instability, as evidenced by the accumulation of single- and double-stranded DNA breaks. However, this does not appear to result in gains or losses of whole chromosomes or chromosomal regions that are occurring extensively and repeatedly enough to be detectable by CGH.

## Discussion

Most of our understanding of RNAPII backtracking and TFIIS-mediated transcript cleavage comes from biochemical and structural studies (Fish and Kane, 2002). However, in yeast cells, most detectable pause sites are associated with backtracked RNAPII complexes (Churchman and Weissman, 2011), and it is therefore important to build on the biochemical insight to understand the mechanisms by which cells deal with backtracked and stopped RNAPII. Here, we used a mutant form of TFIIS (TFIIS_mut_) to study the consequences of trapping RNAPII in an inactive, paused, or backtracked state in human cells. Our study provides new insight into the effect on RNAPII transcript elongation when transcript cleavage is inhibited, as well as important new information on R-loop biology. Most notably, we find that increased RNAPII pausing and backtracking results in R-loop formation and genome instability, providing a direct link between transcription stress, R-loop formation, and DNA damage.

Expression of TFIIS_mut_ in human cells affects transcript elongation and cell viability, in agreement with our previous results in yeast (Sigurdsson et al., 2010). Clear evidence for an average transcriptional slow-down is observed, including a relative depletion of RNAPII toward the end of genes and effects on co-transcriptional processing, including mRNA splicing and termination. At the level of gene expression, this has the consequence that long genes are more negatively affected, with the average length of the downregulated genes being almost 200 kb, while that of the upregulated genes is less than 20 kb. Taken together, our data thus support a crucial role for transcript cleavage in supporting transcript elongation in human cells. These results complement recently published data by the Bentley laboratory using the same TFIIS mutant (Sheridan et al., 2019).

During backtracking, the active site of RNAPII loses control of the RNA 3′ end, which is extruded through a channel below the active site (Cheung and Cramer, 2011, Wang et al., 2009). Transcription problems resulting in backtracking will occur in a stochastic fashion across the genome, but due to the existence of TFIIS and intrinsic transcript cleavage by RNAPII, such backtracks are, on average, likely to be relatively short-lived. Backtracks are thus individually random and rare but statistically predictable and globally very frequent. This means that studying the consequence of RNAPII retrograde motion inside cells is challenging. However, biochemical reconstitution experiments have taught us important, basic properties of RNAPII backtracking. In the experiments described here, RNAPII was incubated for extended periods of time in the absence of nucleotides to allow a small proportion of elongation complexes to backtrack the considerable distance required for anterior R-loops to be able to form. However, during events such as head-to-tail collision between RNAPIIs, backtracking for a distance of more than 25 nt is almost instantaneous (Saeki and Svejstrup, 2009), and despite the fact that RNAPII possesses intrinsic cleavage activity, it fails to recover efficiently from backtracks on its own when they are beyond ∼10 nt (Lisica et al., 2016). In general, RNAPII ceases to transcribe and is unable to recover from backtracks at only one-third of the force determined for *E. coli* RNAP (Galburt et al., 2007), suggesting that the eukaryotic polymerase that transcribes protein-coding genes is intrinsically obstacle sensitive and prone to get trapped after retrograde motion. It is thus easy to imagine that if pausing and backtracking is not dealt with quickly inside cells, then protracted backtracking may take place and the lengthening, exuded RNA might form an R-loop anterior to the RNAPII complex. We believe that this situation may hitherto have been overlooked when considering the genesis and consequences of R-loops, which have invariably been depicted as occurring to the posterior side of RNAPII (Figure 5E).

While extensive transcription stress may give rise to some RNAPII dissociation or degradation, potentially allowing the nascent transcript to invade the DNA template, the results presented here support the idea that anterior R-loops can accumulate in direct response to transcriptional backtracking. Our results also indicate that R-loops resulting from transcription stress give rise to genome instability, including DNA strand breaks. Interestingly, CGH analysis failed to uncover gains or losses of whole chromosomes or chromosomal regions resulting from these DNA breaks. It is presently unclear whether the stochastic nature of RNAPII backtracking means that any gains or losses are not occurring extensively and repeatedly enough in the same regions to be detectable by CGH or if the strand breaks occurring in response to R-loop formation in TFIIS_mut_-expressing cells are simply (invariably) precisely repaired.

Previous work indicated that genome instability observed after depletion of factors such as the THO complex, splicing factor SRSF1, DDX23, SETX, DHX9, TOP1, and many others is caused by R-loops as well (see, for example, Domínguez-Sánchez et al., 2011, Groh et al., 2017, Huertas and Aguilera, 2003, Li and Manley, 2005, Manzo et al., 2018, Skourti-Stathaki et al., 2011, Sridhara et al., 2017, Tuduri et al., 2009). Defects in these factors have been presumed to result in R-loops posterior to RNAPII, but whether and how these R-loops relate to those caused by TFIIS_mut_ expression remains to be investigated. Interestingly, while it was previously shown in *E. coli* that DSBs accumulate at sites of co-directional collisions between the replisome and backtracked elongation complexes (Dutta et al., 2011), recent studies aimed at addressing the molecular basis of transcription-replication collisions and their relationship with genome-destabilizing R-loops concluded that head-to-head collision of the DNA replication fork with DNA-RNA hybrids constitutes a particular threat in human cells, bacteria, and yeast (García-Rubio et al., 2018, Hamperl et al., 2017, Lang et al., 2017). We suggest that the consequence of R-loop-mediated transcription-replication conflicts may depend on whether the R-loops in question are anterior or posterior to the RNAP.

## STAR★Methods

### Key Resources Table

**Table:** 

REAGENT or RESOURCE	SOURCE	IDENTIFIER
**Antibodies**		

Monoclonal to RNAPII phosphorylated CTD (4H8)	The Francis Crick Institute Core Facility	N/A
Monoclonal to CTD repeat RNAPII (8WG16)	The Francis Crick Institute Core Facility	N/A
Mouse monoclonal to S9.6	The Francis Crick Institute	N/A
Polyclonal to N-terminal TFIIS (TCEA1)	This paper	N/A
Polyclonal to total RNAPII (N-20)	Santa Cruz	Sc-899; RRID: AB_632359
Monoclonal to Flag	Sigma	F1804; RRID: AB_262044
Monoclonal to Flag	Sigma	F3165; RRID: AB_259529
Polyclonal to Histone H2Ax	Abcam	ab11175; RRID: AB_297814
Histone gH2Ax	Abcam	ab2893; RRID: AB_303388
Monoclonal to Vinculin	Sigma	V9131; RRID: AB_477629
Monoclonal to ssDNA	Milipore	MAB3031; RRID: AB_10677396
Streptavidin-HRP	Pierce	21130;
Polyclonal to 53BP1	Abcam	ab36823; RRID: AB_722497
Polyclonal to 53BP1	Novus Biologicals	NB100-304; RRID: AB_10003037
Monoclonal to Ubiquitin (P4D1)	Cell Signaling	3936S; RRID: AB_10691572
Secondary Goat Anti-Mouse Alexa Fluor 594	ThermoFisher Scientific	R37121; RRID: AB_2556549
Secondary Chicken Anti-Mouse Alexa Fluor 488	ThermoFisher Scientific	A21200; RRID: AB_141606
Secondary Goat Anti-Rabbit Alexa Fluor 647	ThermoFisher Scientific	A21244; RRID: AB_141663
Li-Cor Secondary Donkey Anti-Rabbit 800CW	Li-Cor	925-32213; RRID: AB_2715510
Li-Cor Secondary Donkey Anti-Mouse 680LT	Li-Cor	926-68022; RRID: AB_10715072
Anti-mouse HRP	Santa Cruz	sc-516102; RRID: AB_2687626
Anti-rabbit HRP	Jackson ImmunoResearch	711-035-152; RRID: AB_10015282

**Bacterial and virus strains**		

NEB® 5-alpha Competent E. coli	NEB	C2988J
Rosetta ™ (DE3) E. coli	Novagen	70954

**Chemicals, Peptides, and Recombinant Proteins**		

5,6-Dichlorobenzimidazole 1-β-D-ribofuranoside (DRB)	Sigma-Aldrich	D1916
RNase H	NEB	M0297S
Superase™	ThermoFisher Scientific	AM2694
Doxycycline	Clonetech	8634-1
MG132	Cayman Chemical	10012628
N-Ethylmaleimide (NEM)	Sigma-Aldrich	E3876
DNase, RNase Free	Promega	M6101
5 Ethynyl-uridine	Jena Bioscience	CLK-N002-10
4-thiouridine	Glentham Life Sciences	GN6085
MTSEA biotin-XX linker (MTSEA Biotincapcap; 2-((6-((6-((biotinoyl)amino)hexanoyl)amino)hexanoyl)amino) ethylmethanethiosulfonate))	Biotium	BT90066
Alexa Flour 488 Azide	ThermoFisher Scientific	A10266
Dynabeads® Protein A/G	ThermoFisher Scientific	10001D/3D
ANTI-FLAG® M2 Affinity Gel	Sigma-Aldrich	A2220, RRID: AB_10063035
3xFLAG peptide	Peptide Chemistry, The Francis Crick Institute	N/A
TCEA1 peptides for N terminal antibody GPSTEKDLDEK KKEPAITSQNSPC-CONH2 and AREESTSSGNVSNRKDE TNARDTC- CONH2	Peptide Chemistry, The Francis Crick Institute	N/A

**Critical Commercial Assays**		

RNeasy Mini Kit	Qiagen	74104
RNA minElute clean-up kit	Qiagen	74204
QiAmp DNA mini kit	Qiagen	51304
μMACS Streptavidin Kit	Miltenyi	130-074-101
TruSeq HT kit	Illumina	20020595
Strand specific TruSeq total RNA kit	Illumina	20020597
KAPA RNA Hyper prep	Illumina	KR1350
Taqman Reverse Transcriptase Reagents	ThermoFisher Scientific	N8080234
Comet Assay Kit	Trevigen	N/A

**Deposited Data**		

Sequencing data	This study	GEO: GSE132400
Mendeley dataset	This study	https://doi.org/10.17632/8hzcg3bk37.1

**Experimental Models: Cell Lines**		

HEK293T-REx™ cell line	Thermo Fischer Scientific	R71007
HEK293T-Rex – TCEA1_mut_	This study	N/A
U2OS	Thermo Fischer Scientific	920022711
U2OS-RNH1	Calderón-Montaño JM (CABIMER)	N/A

**Experimental Models: Organisms/Strains**		

**Recombinant DNA**		

pcDNA4/TO (empty plasmid)	ThermoFisher Scientific	V102020
pET28aSUMO (empty plasmid)	Kind gift from Peter Cherepanov	N/A
pcDNA4/TO-TCEA1_mut_ cDNA	This paper	N/A
pET28aSUMO-TCEA1_WT_ cDNA	This paper	N/A
pET28aSUMO-TCEA1_mut_ cDNA	This paper	N/A

**Sequence-Based Reagents**		

See Table S4		N/A

**Software and Algorithms**		

SAMtools	Li et al., 2009	http://samtools.sourceforge.net/
BWA	Li and Durbin, 2009	http://maq.sourceforge.net/
BEDtools	Quinlan and Hall, 2010	https://bedtools.readthedocs.io/en/latest/
MISO	Katz et al., 2010	http://genes.mit.edu/burgelab/miso/
DEXSeq	Anders et al., 2012	http://bioconductor.org/packages/release/bioc/html/DEXSeq.html
STAR version 2.5.2a	Dobin et al., 2013	https://github.com/alexdobin/STAR
RSEM 1.2.31	Li and Dewey, 2011	https://github.com/deweylab/RSEM
DESeq2	Love et al., 2014	N/A
DAVID Bioinformatics resource	Huang et al., 2009a, Huang et al., 2009b	https://david.ncifcrf.gov/
HCS Studio™ 2.0 Cell Analysis Software	ThermoFisher Scientific	https://www.thermofisher.com/order/catalog/product/SX000041A

**Other**		

High glucose DMEM	ThermoFisher Scientific	11965118
Tet-free FBS	Gibco	16000-044
Poly-lysine	Sigma Aldrich	P7280
VECTASHIELD Antifade Mounting Medium containing DAPI	Vector Laboratories	H-1200
ProLong Gold Antifade Mounting Medium	ThermoFisher Scientific	P36930
3-8% Tris Acetate gels	Bio-Rad	3450130
4-15% TGX gels (18 wells/26 wells)	Bio-Rad	56711084/5
10% TBE-Urea gels	Novex	EC68755BOX
15% TBE-Urea gels	Novex	EC6885BOX
Complete EDTA-free protease inhibitor cocktail	Sigma Aldrich	05056489001
Nitrocellulose membrane	GE Healthcare Life Sciences	1060002
Amersham Hybond N+ membrane	GE Healthcare Life Sciences	RPN203B
PhosSTOP™	Sigma-Aldrich	4906837001
Benzonase	MerckMillipore	70746-4
iTaq™ Universal SYBR Green Supermix	Bio-Rad	172-5124
Lipofectamine 2000	Thermo Fischer Scientific	11668019
Proteinase K	Sigma Aldrich	3115887001
AMPureXP beads	Beckman Coulter	A63881
T4 Polynucleotide Kinase	ThermoFisher Scientific	EK003
TRIzol™ Reagent	ThermoFisher Scientific	15596026
Phenol/Chloroform pH 4.5	ThermoFisher Scientific	AM9722
Dynabeads™ MyOne™ Streptavidin C1	ThermoFisher Scientific	65001
Decade™ RNA markers system	ThermoFisher Scientific	AM7778
^32^ P-alpha ATP	Perkin Elmer	NEG003H250UC
^32^ P-gamma ATP EasyTide Lead	Perkin Elmer	NEG502A250UC
HiTrap ™ Heparin SP FF column (1ml)	GE Healthcare	17505401
Fast Flow Q Sepharose	GE Healthcare	17051005
Amicon Ultra-15 15K MWCO spin concentrators	MerckMillipore	UFC900308

### Lead Contact and Materials Availability

Further information and requests for resources and reagents, such as plasmids, should be directed to and will be fulfilled by the Lead Contact, Jesper Svejstrup (jesper.svejstrup@crick.ac.uk).

### Experimental Model and Subject Details

#### Cell Lines and Culture Conditions

Human HEK293T-REx (Invitrogen) cells were grown at 37°C, 5% CO_2_ in DMEM supplemented with 10% fetal bovine serum and 5% penicillin/streptomycin. The Francis Crick Institute Cell Services department screened cell lines for mycoplasma contamination, and authenticated species by STR profiling and PCR based analysis.

U2OS and U2OS-RNH1 (Tet-On system, Invitrogen) cells were maintained in DMEM medium, supplemented with 10% heat-inactivated fetal calf serum (FCS) and culture at 37°C, 5% CO_2_. All cell lines have been confirmed to be mycoplasma-free.

### Method Details

#### Generation of Stable Cell Line

For generation of stable doxycycline-inducible TFIIS_mut_ cell lines, HEK293T-REx were transfected with pcDNA4/TO-TFIIS_mut_ plasmid, expressing full length mutant TFIIS (TCEA1) under a tetracycline/doxycycline responsive promoter, and Zeocin-selected.

#### Colony Formation Assay

Cells stably expressing dox-inducible TFIIS_mut_ were seeded at a low density (200 cells/well) into 6-well plates n in the absence or presence of doxycycline. Colonies were allowed to form over a 10-14 day period after which they were fixed by 4% (v/v) formaldehyde and stained with 0.1% (w/v) crystal violet. Colonies from two biological replicates (each seeded into triplicate wells) were counted.

#### Immunofluorescence

Cells were grown onto poly-lysine (Sigma-Aldrich P7280) coated coverslips, fixed in 4% (v/v) formaldehyde and processed as previously described (Kantidakis et al., 2016). Briefly, the primary antibody, used in 1:1000 dilution was anti 53BP1 (Abcam, ab 36823) and the secondary antibody anti-rabbit Alexa Fluor 594 (Life Technologies). The coverslips were mounted using Vectashield Antifade Mounting Medium containing DAPI (Vector Laboratories, H-1200) and visualized using a Zeiss fluorescent microscope with a 63x/1.4 oil immersion and quantified with ImageJ.

For R-loop detection, cells were grown on coverslips, fixed and permeabilized in 100% ice cold methanol and acetone for 10 min and 1 min on ice, respectively, and processed as previously described (Sridhara et al., 2017). Briefly, the primary antibody, S9.6 was used in 1:500 dilution, and the secondary antibody anti-mouse Alexa Fluor 594 (Life Technologies). The coverslips were mounted using Vectashield Antifade Mounting Medium containing DAPI (Vector Laboratories, H-1200) and visualized using a Zeiss fluorescent microscope with a 63x/1.4 oil immersion and quantified with ImageJ.

#### 53BP1 Foci Analysis in U20S

For 53BP1 foci analysis in U2OS and U2OS-RNH1 (the latter a cell line stably integrating a Tet-on system to induce overexpression of RNase H1 (kindly provided by Calderón-Montaño JM)), cells were plated onto coverslips and after 24 hours were transfected using Lipofectamine 2000 with a plasmid to overexpress Flag-tagged TFIIS_mut_ or with a plasmid to overexpress luciferase as a mock control. Before transfection, the medium was replaced with fresh medium containing, or not, 5 μg/ml of doxycycline to induce RNase H1 overexpression in U2OS-RNH1 cells. Cells were fixed at 48 hours after transfection with 3.7% formaldehyde (v/v) in PBS for 15 minutes, washed 4 times with PBS, permeabilized with 0.5% Triton X-100/PBS for 5 minutes and blocked with 3% bovine serum albumin (BSA) in PBS for 1 hour. The coverslips were then incubated with anti-53BP1 (Novus Biologicals) and anti-Flag (Sigma Aldrich) antibodies diluted 1:500 in 3% BSA/PBS for 2 hours followed by 3 washes with PBS and 1 hour incubation with 1:1000 diluted secondary antibodies conjugated with Alexa Fluor 488 (chicken anti-mouse) and Alexa Fluor 647 (goat anti-rabbit) (Invitrogen, cat.no A21200 and A21244 respectively). After 2 washes with PBS, nuclei were counterstained with 10 μg/ml of DAPI in PBS for 5 minutes, washed 3 more times and mounted with ProLong Gold antifade reagent (Invitrogen). Random images were acquired with a 63X objective using a Leica DM6000 wide-field microscope. Data acquisition and image processing were performed using the LAS AF software (Leica). Microscopy data analysis was performed using the Metamorph v7.5.1.0 software (Molecular Probes).

#### Alkaline Comet Assay

U2OS and U2OS-RNH1 cell lines were transfected as detailed above. Following 48 hours after transfection and RNase H1 induction, comet assay was performed with a commercial kit (Trevigen, Gaithersburg, MD, USA) according to the manufacturer’s instructions. Comet slides were stained with SYBR Green, and images were captured at 10X magnification using a Leica DM6000 wide-field microscope. Comet tail moments were analyzed using TriTek CometScore Professional (version 1.0.1.36) software. At least 100 cells were scored in each experiment to calculate the median of the tail moment.

#### Western Blotting

For whole cell extracts, cell pellets were lysed in TENT cell lysis buffer (50 mM Tris-HCl pH 7.5, 150 mM NaCl, 2 mM EDTA, 0.5% Triton X-100, PhosSTOP™ (Sigma-Aldrich 04906837001) and Protease Inhibitor Cocktail). 40 μg protein/lane was separated by SDS-PAGE on 4%–12% polyacrylamide gels and transferred onto nitrocellulose membrane (GE Healthcare Life Sciences 10600002). Membranes were blocked in 5% (w/v) skimmed milk in PBS-T (PBS 0.05% (v/v) Tween20) for 30 min are room temperature and incubated with primary antibody in blocking buffer overnight at 4°C. Primary antibodies are listed in Key Resources Table. Membranes were washed several times in PBS-T, incubated with Li-Cor fluorescent dye-conjugated secondary antibodies or HRP-conjugated secondary antibodies in blocking buffer and visualized.

#### Flag- and RNAPII-Immunoprecipitation – Extract Preparation

TFIIS_mut_ cells were cultured in the absence or presence of doxycycline for 48 hours. Phosphatase inhibitors (PhosSTOP™, Sigma Aldrich) and Protease inhibitors (Sigma Aldrich) were added fresh to all buffers. A ∼0.8 mL cell pellet volume was resuspended in 2 pellet volumes (PVs) of hypotonic buffer (HB) buffer (10 mM HEPES pH 7.5, 10 mM KCl, 1.5 mM MgCl_2_). Cells were homogenized in a Dounce homogenizer with 10 strokes loose pestle/pestle A and incubated on ice for 15 min. Nuclei were pelleted by centrifugation at 3900 rpm for 15 min. The supernatant was removed and supplemented with glycerol to a final concentration of 0.05%, NaCl to a final concentration of 150 mM, and EDTA for a concentration of 3 mM. After centrifugation at 20000 g for 10 min, this cleared cytoplasmic fraction was kept for further analysis. The nuclei in the pellet were resuspended in 2 PVs of nucleoplasmic extraction buffer (20 mM HEPES pH 7.9, 1.5 mM MgCl_2_, 150 mM KCH_3_COO, 10% glycerol, 0.05% NP-40). Nuclei were lysed by 15 strokes loose pestle/pestle A and incubated on ice for 20 min followed by centrifugation at 20,000 g for 20 min. The nucleoplasmic supernatant was pooled with the cytoplasmic fraction and kept aside as the ‘soluble fraction’. The chromatin pellet was resuspended in 1 mL chromatin digestion buffer (20 mM HEPES pH 7.9, 1.5 mM MgCl_2_, 10% glycerol, 150 mM NaCl, 0.1% NP-40, 250 U benzonase/ml), incubated 45 min on ice and centrifuged for 20 min at 20,000 g. The supernatant was removed and kept as ‘low salt chromatin fraction’. The remaining pellet was resuspended in 0.6 PV of 500 mM NaCl buffer (20 mM HEPES pH 7.9, 3 mM EDTA, 1.5 mM MgCl_2_, 10% glycerol, 500 mM NaCl, 0.1% NP-40) and incubated 20 min on ice. 1.4 PV of salt dilution buffer (20 mM HEPES pH 7.9, 3 mM EDTA, 1.5 mM MgCl_2_, 10% glycerol, 0.1% NP-40) was added and centrifuged for 15 min at 20,000 g. The supernatant was pooled with the low salt chromatin fraction and used for further affinity purifications.

#### Flag (M2)-Affinity Purification of TFIIS

The chromatin fractions were incubated with approximately 30 μL pre-equilibrated M2 anti-FLAG agarose beads (Sigma), rotating at 4°C overnight. After incubation, the flow-through was collected and the beads were washed with 40 CV IP wash buffer (20 mM HEPES pH 7.9, 1.5 mM MgCl_2_, 150 mM NaCl, 0.05% NP-40, 1x Protease inhibitor mix, PhosphoStop phosphatase inhibitor). Immunoprecipitates were eluted using 500 μg/ml 3x FLAG peptide (The Francis Crick Institute), dissolved in IP wash buffer, by incubation for 30 min at 4°C . 30 μL of elution fractions were subjected to SDS-PAGE and subsequently stained with Instant Blue Coomassie. The stained proteins were excised from the polyacrylamide gel, cut into 8 equal slices and submitted to mass spectrometry analysis. Proteins were in-gel digested with trypsin using a Janus Automated Workstation (Perkin Elmer) and peptides were analyzed using a LTQ Orbitrap-Velos mass spectrometer coupled to an Ultimate3000 HPLC and equipped with an EASY-Spray nanosource (ThermoFisher Scientific).

#### RNAPII Affinity Purification

The chromatin fractions were incubated with approximately 30 μL pre-equilibrated magnetic Dynabeads Protein A (Invitrogen) coupled with 1 μg of RNAPII antibody (4H8) rotating at 4°C overnight. After incubation, the flow-through was collected and the beads were washed with 50 CV wash buffer (20 mM HEPES pH 7.9, 1.5 mM MgCl_2_, 150 mM NaCl, 0.05% NP-40, 1x Protease inhibitor mix, PhosphoStop phosphatase inhibitor). Proteins (30 μL of elution fractions) were eluted by boiling in Laemmli loading buffer for 5 minutes at 95°C, before being subjected to SDS-PAGE and subsequently stained with Instant Blue Coomassie. The stained proteins were excised from the polyacrylamide gel, cut into 8 equal slices and submitted to mass spectrometry analysis. Proteins were in-gel digested and subjected to mass spectrometry as above. The Proteomics Laboratory at Francis Crick Institute performed all mass spectrometry analyses described in this study.

#### DSK2-Pulldown

DSK2 resin was prepared as previously described (Anindya et al., 2007). Briefly, Pull-down of ubiquitylated proteins was performed by incubating 25 μL DSK2 resin with 500 μg WCE in 500 μl overnight at 4°C. The beads were washed 4 times with 1ml TENT buffer (50 mM Tris-HCl pH 7.5, 150 mM NaCl, 2 mM EDTA, 0.5% Triton X-100) for 5 min each. Afterward, the beads were resuspended in 100 μL 4xSDS loading buffer and boiled for 10 min at 95°C. 15 μL of sample was used for SDS-PAGE and immunoblotting.

#### 5-Ethynyl Uridine (EU) Transcription Assay

Cells were incubated for 48 hr with or without doxycycline. Media was replaced with fresh media containing 0.75 mM 5-Ethynyl uridine (EU) and cells were incubated for different amount of time (30min, 60min, 90 min, 120 min, 180 min). EU-containing media was removed and cells were fixed in PBS-buffered formaldehyde (3.7%) for 45 min at room temperature, washed once with PBS using a plate washer, followed by permeabilization with 0.5% TX-100 diluted in PBS for 30 min. Permeabilized cells were washed once with PBS, and then Alexa Fluor 488 Azide fluorophores were covalently attached to the EU-containing nascent RNA by click reaction (100 mM Tris pH 8.5, 4 mM Cu_2_SO_4_, 10 μM Alexa azide 488, 100 mM Ascorbic Acid) for 1 hr at room temperature. Cells were washed 3 times in 100 mM Tris, pH 7.5 and stained with DAPI (4′,6-diamidino-2-phenylindole dihydrochloride) at a final concentration of 1 μg/ml. Cells were washed once with PBS. Automated image acquisition of 6 fields per well was performed (Cellomics Array Scan VTI, ThermoFisher Scientific) using a 10X objective.

Image analysis was performed using HCS Studio 2.0. Cell nuclei were masked using the DAPI staining. The average intensity of Alexa Fluor 488-conjugated EU-labeled RNA was measured for each nucleus in at least 3 separate wells and plotted.

#### ChIP-Sequencing

For chromatin-immunoprecipitation (ChIP) experiments, cells were harvested by trypsin-treatment and fixed in suspension with formaldehyde, 1% final concentration, for 10 min at room temperature, with rotation. The crosslinking reaction was quenched with glycine (125 mM final concentration) for 5 min. Cells were washed twice with ice-cold 1 × PBS and lysed in 1 mL of ChIP cell lysis buffer (5 mM HEPES pH 8.0, 85 mM KCl, 0.5% NP-40, and protease inhibitors) and incubated 5 min on ice. Nuclei were pelleted by centrifugation at 3,900 g for 5 min at 4°C. Finally, nuclei were lysed in ChIP nuclear lysis buffer (50 mM Tris-HCl pH 8.1, 10 mM EDTA (pH 8.0), 1% SDS, and protease inhibitors) and incubated 5 min on ice. Nuclear lysate was sheared by using an ice-water bath-embedded Bioruptor sonication system at high power, 30 s on, 30 s off mode for 5–10 min. The size of the sheared DNA was checked by 2% agarose gel electrophoresis to be between 300–600 base pairs (bp). Sonicated chromatin was cleared by centrifugation at 20,000 g for 15 min at 4°C. Before the immune-precipitation, chromatin was diluted 1:5 with ChIP dilution buffer (0.01% SDS, 1.1% Triton X-100, 1.2 mM EDTA (pH 8.0), 16.7 mM Tris-HCl pH 8.1, 167 mM NaCl and protease inhibitors). 1 μg of RNAPII antibody (4H8; recognizes all forms of RNAPII), or 1 μg of mouse IgG (Sigma), was bound to 15 μL of Protein A Dynabeads (Invitrogen) in 200 μL 5% BSA in PBS for 1 hr (h), before being washed twice with 500 μL of the same buffer. The sonicated chromatin was incubated with the antibody-conjugated beads overnight (o/n) at 4°C with rotation. Beads were washed twice with 1 mL of each of the following buffers: ChIP low salt buffer (0.1% SDS, 1% Triton X-100, 2 mM EDTA, 20 mM Tris-HCl pH 8.1, 150 mM NaCl); ChIP high salt buffer (0.1% SDS, 1% Triton X-100, 2 mM EDTA, 20 mM Tris-HCl pH 8.1, 500 mM NaCl); and ChIP LiCl buffer (10 mM Tris-HCl pH 8.0, 250 mM LiCl, 1% NP-40, 1% deoxycholic acid, and 1 mM EDTA). Beads were washed once with 1 mL of TE buffer (pH 8) and centrifuged for 1 min at 14,000 g before removing the buffer. Beads were finally suspended in 40 μL Elution Buffer (50 mM Tris-HCl pH 8.0, 10 mM EDTA, 1% SDS) and incubated at 65°C for 15 min. The eluted ChIP material was incubated at 65°C o/n to revert the crosslinking with an additional 90 μL of TE 1 × /SDS1% and 1 μL 10 mg/ml RNase A. In parallel, the whole-cell extract was also RNase treated and reverse crosslinked o/n at 65°C. Proteinase K (100 μg) and Glycogen (20 μg) were added to the eluted ChIP material and incubated for 2h at 37°C, and DNA was twice extracted with phenol-chloroform-alcohol isoamylic acid, and precipitated with ethanol/NaCl. Precipitated DNA was submitted for further manipulation by standard ChIP-seq library preparation techniques (Illumina) and Advanced Sequencing on an Illumina HiSeq 2500 DNA sequencer. 51 bp single-end reads were Illumina adaptor trimmed using cutadapt 2.7.12 (-e 0.1 –a AGATCGGAAGAGC –q 20,20) to a minimum length of 30bp prior to alignment to the GRCh38 genome assembly using BWA mem 0.12.7 (Li and Dewey, 2011) with default settings. BAM files were sorted and indexed using Picard. Further analysis was conducted using Bioconductor (Gentleman et al., 2004).

#### mRNA-Sequencing

Cell were induced for 48 hours before RNA was extracted using RNeasy Kit (QIAGEN 74104) according to the manufacturer’s instruction including the on-column DNase treatment. 2 μg total RNA was used for purification and analyzed on a 2100 Bioanalyzer (Agilent Technologies). All samples had an RIN value of greater than 8. The purified RNA was used for the preparation of Poly(A)-selected mRNA libraries using the TruSeq RNA sample preparation kit and sequenced on an Illumina HiSeq 4000 as 101bp paired-end reads.

#### TT-Seq (Nascent RNA-Seq)

TFIIS_mut_ cells were cultured for 48 hours in the absence or presence of doxycycline. 4-thioridine (4SU) was added directly to the tissue culture media to a final concentration of 1 mM, for 20 min. The reaction was stopped by removing the media and 1ml of TRIZOL reagent was added on top of the cells, followed by Phenol/Chloroform/Isoamyl alcohol (25:24:1) (Thermo Fisher Scientific) purification. RNA was precipitated by addition of 1.1 volume of isopropanol and incubated at room temperature (RT) for 20 min. The pellet was resuspended in 100 μL RNase-free water and the RNA concentration was measured using Qubit BR RNA assay. 100 μg of human RNA was mixed with 1 μg *S. cerevisiae* spike-in RNA (strain BY4741, MATa, his3D1, leu2D0, met15D0, ura3D0) 4-thiouracil (4TU)-labeled RNA, in a total volume of 100 μl. In order to fragment the RNA, 20 μL of 1M NaOH was added and incubated 20 min on ice. Fragmentation was stopped by addition of 80 μL 1M Tris pH 6.8 and the reaction was cleaned by running it twice on Micro Bio-Spin™ P-30 Gel Columns (BioRad 732-6223) spin columns according to the manufacturer’s instructions. The RNA was biotinylated by adding MTSEA biotin-XX linker (Bioutium, BT90066) and incubating the reaction for 30 min at RT in the dark. The biotinylated RNA was Phenol/Chloroform/Isoamyl alcohol purified, followed by ethanol-precipitating the RNA. 4SU incorporation was measured by dropping 5 μL of diluted RNA (2:5) onto a N+ membrane (GE Healthcare Life Sciences), UV crosslinked twice at 2000 μJ and blocked for 20 min at RT in blocking buffer (10% SDS, 1mM EDTA in 1xPBS). Membrane was probed with 1:50,000 dilution of 1 mg/ml streptavidin-horseradish peroxidase (Pierce) in blocking solution for 15 min. The membrane was washed six times in PBS, containing decreasing concentrations of SDS (10%, 1% and 0.1%, each applied twice). The signal of biotin-bound HRP was visualized by ECL detection.

4SU biotinylated RNA was further purified using the μMACS Streptavidin MicroBeads (Miltenyi, 130-074-101). Labeled RNA was eluted by the addition of 100 μL freshly prepared 100 mM DTT, followed by a second elution round 5 min later. RNA was recovered by using the RNeasy MinElute Spin columns (QIAGEN, 74204). RNA was eluted in 15 μL RNase free water and 1 μL was analyzed on a 2100 Bioanalyzer with the mRNA pico settings (Agilent Technologies). cDNA libraries were prepared from the purified RNA by using the KAPA RNA Hyper Prep kit and sequenced on an Illumina HiSeq 4000 as 76 bp single-end reads.

#### Reverse Transcriptase Quantitative PCR

RNeasy Mini Kit (QIAGEN, 74104)-purified RNA was used to generate random hexamer primed cDNA libraries using Taqman Reverse Transcriptase Reagents (Thermo Fischer Scientific, N8080234). Quantitative PCR were performed using iQ SYBR green Mastermix, 0.3 μM primer concentration and 1 μL of cDNA library per reaction. Reference gene-normalized RNA expression was compared between TFIIS_mut_ and parental samples using the Livak equation (Livak and Schmittgen, 2001). For example, to measure the expression of different genes, the following equation was used (2^∧^-((CT_gene_-CT_GAPDH_)_TFIISmut_-(CT_gene_-CT_GAPDH_)_Parental_)). Primer sequences are found in Table S3.

#### DNA-RNA Slot Blot

Genomic DNA was isolated by using the QIAmp DNA Mini kit (QIAGEN, 51304). Genomic DNA (175ng) was treated with 2U of RNase H (NEB, M0297S) per μg of DNA for 2 hours at 37°C before loading on the slot blot and transferred onto a N+ membrane. Following UV crosslinking at 1200 μJ, membranes were blocked in 5% Milk/PBS-Tween (0.05% Tween20) and incubated overnight with S9.6 antibody (1:1000). After the last wash, the membrane was incubated for 1 hour at RT with secondary antibody (goat anti mouse horseradish peroxidase). After washing three times for 10 min with PBS-Tween (0.05% Tween20), the membrane was briefly dried and incubated with SuperSignal West Pico chemiluminescent substrate (Thermo Fischer Scientific) for 2 minutes. The chemiluminescent signal was detected by exposure of the membrane to Amersham Hyperfilm ECL (GE Healthcare). To determine the loading, the membrane was washed twice for 10 min in PBS-Tween (0.05% Tween20) and denatured with 0.5N NaOH, 1.5M NaCl for 10 min at RT, followed by 10 min incubation with 1M NaCl, 0.5 M Tris pH 7. After one wash with PBS-Tween (0.05% Tween20) the membrane was incubated for 2 hours at RT with ssDNA antibody (Milipore, MAB3031), and detection was carried out as above.

#### DNA-RNA Hybrid Mapping (DRIP)

DRIP-qPCR experiments were performed similar to the protocol described in (Sridhara et al., 2017) with the following modifications. Briefly, nucleic acids were extracted using standard phenol-chloroform extraction and re-suspended in DNase/RNase-free water. Nucleic acids were fragmented using a restriction enzyme cocktail (20U each of EcoRI, BamHI, HindIII, BsrgI and XhoI (NEB)). Half of the sample was digested with 40U RNase H (NEB) to serve as negative control, for 24 hours at 37°C. Digested nucleic acids were cleaned with phenol-chloroform extraction and re-suspended in DNase/RNase-free water. RNA-DNA hybrids were immunoprecipitated from total nucleic acids using 5 μg of the S9.6 antibody in binding buffer (10 mM NaPO_4_, pH 7, 140 mM NaCl, 0.05% Triton x-100) overnight incubation at 4°C with 50 μL protein A/G magnetic beads (Thermo Fischer Scientific). Isolated complexes were washed three times with binding buffer and once with TE buffer for 10 min at RT, before elution with 500 μl of elution buffer (50 mM Tris pH 8, 10 mM EDTA, 0.5% SDS). Proteinase K digestion was performed for 30 min at 55°C, followed by phenol-chloroform extraction as before. Pellet was air-dried and resuspended in 100 μL DNase/RNase free water. The DNA was further used to determine the relative occupancy of the immunoprecipitated DNA-RNA hybrid by RT-qPCR and analyzed with the primers described in Table S4.

#### Recombinant Protein Expression and Purification

Rosetta™ BL-21 (DE3) *E.coli* cells were transformed with expression plasmid pET28aSUMO-TFIIS_WT_ or pET28aSUMO-TFIIS_mut_ and selected with 50 μg/ml kanamycin. 1L *E.coli* cultures were grown to an optical density (OD_600nm_) of 0.6. TFIIS protein expression was induced with 0.5 mM IPTG and the culture was shifted to 30°C for 4 hours. Cell pellets were resuspended in 50 mL lysis buffer (25 mM HEPES pH 7.9, 250 mM NaCl, 0.4% Triton X-100, 10% glycerol, 1 mM DTT,15 mM imidazole, 10 μM ZnCl) and left for 20 min on ice. Extracts were sonicated for 2 min at 30% amplitude, in 5 s bursts on ice, followed by ultracentrifugation at 45K for 30 min at 4°C in a 70Ti rotor (Beckman Coulter). 4 mL Nickel NTA resin was washed with water by using a peristaltic pump and then with lysis buffer to equilibrate (5 CV at 1 ml/min). Lysates were loaded at 0.5 ml/min in the cold room, followed by washing with 40 mL lysis buffer at 1 ml/min. Proteins were eluted with 25 mL elution buffer (25 mM HEPES pH 7.9, 10% glycerol, 500 mM NaCl, 1 mM DTT, 500 mM imidazole, 10 μM ZnCl) at 0.5 ml/min. Load, flow-through and elutions were resolved by running on an SDS-PAGE gel and stained with Instant Blue Coomassie. Following addition of 7 μL of Ulp1 (22 mg/mL) to remove the SUMO tag, the eluates (25 ml) were dialyzed (3.5 kD MWCO, diameter 29 mm) overnight at 4°C against 1L of dialysis buffer (100 mM NaCl, 10% glycerol, 25 mM HEPES pH 7.9, 10 μM ZnCl, 1 mM DTT). The dialyzed sample was further purified by loading it via a 50 mL Superloop (GE Healthcare) onto a 1mL HiTrap Heparin SP FF column (GE Healthcare). Fractions were eluted with a gradient from 100 to 400 mM NaCl in 0.5ml fractions and resolved by SDS-PAGE gel as before. Fractions containing TFIIS were pooled and concentrated to a volume of approximately 4mL using an Amicon® Ultra-15 15K MWCO spin concentrators (Millipore).

Yeast proteins were prepared as described in (Sigurdsson et al., 2010).

#### Purification of Bovine Pol II

Bovine RNAPII was prepared as previously described (Hu et al., 2006) with modifications. Unless otherwise noted, all steps were completed at 4 °C. Calf thymus was homogenized for 3 min in buffer A (50 mM Tris, pH 7.9 at 4 °C, 10 μM ZnCl_2_, 10% glycerol, protease inhibitors (Sigma Aldrich)) using a 2L blender (Waring). The homogenized material was centrifuged and the supernatant filtered through two layers of Bioprep nylon filter cloth. A 10% solution of polyethyleneimine, pH 7.8 at 25 °C, was added to a final concentration of 0.05%, and the material was stirred for 30 min, then centrifuged for 30 min at 12,000*g*. The resulting pellets were re-dissolved in buffer B (50 mM Tris, pH 7.9 at 4 °C, 10 μM ZnCl_2_, 10% glycerol, 150 mM (NH4)_2_SO_4_, protease inhibitors (Sigma Aldrich)). After centrifugation, the supernatant was loaded on a 120 mL Fast Flow Q Sepharose (GE Healthcare) column, equilibrated in buffer B, by using a peristaltic pump at 5 ml/min. The column was washed with three column volumes of buffer B, followed by step-elution with buffer C (50 mM Tris, pH 7.9 at 4 °C, 10  μM ZnCl_2_, 500 mM (NH4)_2_SO_4_, protease inhibitors). The material was further purified using a 5 mL gravity flow column of 8WG16 (αRPB1 CTD) antibody-coupled Sepharose. The input was loaded overnight using a peristaltic pump at 0.1 ml/min. After application of the input material, the antibody column was washed with ten column volumes of buffer C, sealed, and allowed to equilibrate to room temperature (20–25 °C) for 15 min. RNAPII was eluted in batch using elution buffer (40% 1,2 propanediol, 500 mM (NH4)_2_SO_4_, 50 mM Tris pH7.8, 10 μM ZnCl_2_), collecting 4x 10 mL fractions at room temperature. The RNAPII-containing fractions were dialyzed against dialysis buffer (50 mM Tris pH 7.8, 10 μM ZnCl_2_, 5 mM DTT, 10% glycerol, 150 mM (NH4)_2_SO_4_) and concentrated using a 100-kDa cut-off Amicon concentrator to a final concentration of 2–4 mg ml^−1^.

#### Oligonucleotides for *In Vitro* Transcription

DNA oligo 1 (transcribed strand):

5′-GAGTTGGTTATGGTAGGTGAGTGTGTGATTGTGTGTTAGTGTGGTGTACCCTTGGGTTCTCTTTTCGCCTTGGGGGCTCCTCCTCCCTCCCTCTTTCCTGATGGCTGTTTGTTTCCTATAGCGTAGGCCTTAGACAATTGCGCATTCAGAC-3′
DNA oligo 2 (non-transcribed strand, biotinylated):
5′-GTCTGAATGCGCAATTGTCTAAGGCCTACGCTATAGGAAACAAACAGCCATCAGGAAAGAGGGAGGGAGGAGGAGCCCCCAAGGCGAAAAGAGAACCCAAGCGACACTTCATTAACACACAATCACACACTCACCTACCATAACCAACTC-3′^∗^
DNA oligo 3 (transcribed strand):
5′-GGCCGGGTAACCCCCGTGTGGAGATGGGTGAGAGATGTTGAGGGCCTGGTCGTTTCCTATAGTTTGTTTCCT-3′
DNA oligo 4 (non-transcribed strand, biotinylated):
∗ 5′-CTAGAGGAAACAAACTATAGGAAACGACCAGGCCCTCAACATCTCTCACCCATCTCCACACGGGGGTTACCCGGCCTGCA-3′
RNA oligo 1 (unlabeled or 5′-FAM labeled):
UUU UUA CAG CCA UC
RNA oligo 2 (5′-FAM labeled):
UGCAUUUCGACCAGGC

#### Transcript Cleavage Experiments

Elongation complexes (ECs) were reconstituted essentially as previously described (Saeki and Svejstrup, 2009) in a step-like manner. Briefly, a DNA-transcribed strand oligo was incubated at 75°C for 5 min, followed by cooling on ice for 5 min. Next, an RNA oligonucleotide was added and incubated for 5 min at room temperature (RT) to allow formation of the DNA/RNA hybrid. 1.5 ug RNAPII was incubated with 2.5 pmol of the DNA/RNA hybrid for 20 min at RT, followed by the addition of 5 pmol of non-transcribed DNA strand (which was typically biotinylated at the 3′ end) and incubation at 37°C for 10 min. Assembled elongation complexes (ECs) were purified by binding to streptavidin beads via biotin on the non-transcribed strand, followed by washing in TB (40 mM KCl, 20 mM Tris pH 7.9, 5 mM DTT, 20 uM ZnCl_2_, 7 mM MgCl_2_). Radiolabeling was carried out by adding P^32^-alpha ATP for 5 min, before starting further transcript elongation. Transcription was performed in TB for 5 min at RT in the presence of 500 μM each of ATP, GTP, UTP up to the first guanine position in the template. After washing away free nucleotides (leading to RNAPII backtracking), ECs were incubated for 30 min with either buffer alone or recombinant TFIIS protein. Reactions were stopped by the addition of stop buffer (20 mM Tris pH 7.9, 0.2% SDS, 50 mM EDTA, proteinase K) followed by incubation at 37°C for 30 min. After phenol/chloroform extraction and ethanol precipitation, samples were resuspended in 95% formamide buffer containing 5 mM EDTA and 0.1%SDS and RNA products were resolved by 8 or 10% denaturing PAGE. Radioactive or FAM labeled bands were visualized by typhoon FLA 9500.

#### *In Vitro* R-Loop Formation Experiments

For R-loop formation experiments, the RNA oligonucleotide was either P^32^-5′ end labeled or 5′-FAM labeled (FAM labeled oligo purchased from IDT) and then annealed to the DNA template strand (DNA oligo 1). For these experiments, 1.5 μg of yeast RNAPII was incubated with 2.5 pmol of the DNA/RNA hybrid, followed by the addition of 5 pmol of the non-template DNA strand (which was biotinylated at the 3′ end), as previously described (Saeki and Svejstrup, 2009). The ECs were immobilized and washed as above. Transcription was performed in TB (40 mM KCl, 20 mM Tris pH 7.9, 5 mM DTT, 20 μM ZnCl_2_, 7 mM MgCl_2_) for 5 min at room temperature (RT) in the presence of 500 μM each of ATP, GTP, CTP up to the first adenine position in the template, generating a 69-nucleotide (nt) product. Unincorporated nucleotides were removed by sequential washes with TB. WT-TFIIS and TFIIS_mut_ were added for 3 min, followed by addition of 5U of RNase H (NEB, M0297S) and incubated at RT for 30 min. For the experiment shown in Figure S4E, the TEC/DNA scaffolds were purified via the biotin-tag on the non-transcribed strand after RNase H treatment to ensure that only anterior R-loops were detected (the label on posterior R-loops is released into the supernatant fraction due to RNase H cleavage). Reactions were stopped by the addition of stop buffer (20 mM Tris pH 7.9, 0.2% SDS, 50 mM EDTA, proteinase K) followed by incubation at 37°C for 10 min. After phenol/chloroform extraction and ethanol precipitation, samples were resuspended in 95% formamide buffer containing 5 mM EDTA and 0.1% SDS and were resolved by 15% denaturing PAGE. Radioactive or FAM labeled bands were visualized by typhoon FLA 9500.

#### N-Terminal TFIIS Antibody Production

An N-terminal TCEA1-specific antibody was raised against a mixture of two peptides:

hsTCEA1 79-101C GPSTEKDLDEKKKEPAITSQNSPC-CONH2
hsTCEA1 103-125C AREESTSSGNVSNRKDETNARDTC-CONH2

Eurogentec were responsible for antibody production, using their 28-days Speedy protocol. Based on the analysis of the pre-immune sera, two rabbits were selected for further immunizations. Each rabbit was injected with 250 μg of the peptide mixture. The final bleeds were analyzed by western blot for specificity and yield

### Quantification and Statistical Analysis

Statistical analysis was performed using GraphPad Prism 6.0e software or Excel Microsoft and the tests described in the figure legends.

#### BigWig Files

Genome coverage bigWig files were generated by converting BAM files to bedGraph format using BEDtools’ (Quinlan and Hall, 2010) genomeCoverageBed function (-bg –split –scale 1) bedGraph files were in turn converted to bigWig format using the bedGraphToBigWig function from the KentTools (Kent et al., 2010) package.

#### mRNA Differential Expression Analysis

Reads were aligned against GRCh38 and Ensembl release 86 transcript annotations using STAR v2.5.2a (Dobin et al., 2013) via the transcript quantification software RSEM 1.2.31 (Li and Dewey, 2011). Resulting genome alignment BAM files were sorted and indexed using Picard 2.1.1. The estimated counts per gene across all samples was used to assess differential expression between parental and mutant cell lines via the R package DESeq2 (Love et al., 2014). An FDR ≤ 0.01 and fold-change of at least ± 2 was used to threshold significance.

#### mRNA-Seq Alternative Isoform Analysis

MISO (Mixture of Isoforms) 0.5.3 was used to identify transcript isoforms differentially regulated between mutant and parental cell lines (Katz et al., 2010). Results were filtered for significance based on a Bayes factor > = 10 and dPSI > = +/−0.2. Additionally, a further filter for an inclusion count of > = 1, exclusion count of > = 1 and a sum of inclusion and exclusion counts > = 10 was applied.

Significant ALE events called in at least 2/3 replicates were selected for further analysis of terminal exon relative expression. A ratio of proximal and distal exon reads-per-kilobase (RPK) was calculated for each mutant and parental sample. The mutant ratios were normalized to the parental ratios and a mean of this score was calculated across replicates. A large positive score indicates a preference for the proximal exon in the mutant sample relative to the parental.

#### TT-Seq Metagene Profiles

Reads were aligned to the GRCh38 genome assembly using STAR 2.5.2a (Li and Durbin, 2009) with default settings. BAM files were sorted and indexed using Picard. ngs.plot software (Katz et al., 2010) was used to generate read coverage profiles over the TSS region −0kb:+120kb of all Ensembl protein coding genes from standard chromosomes (n = 19,919) with the following settings: -L 2000 –F chipseq –R bed. A subset of genes > = 60kb in length was used to create similar profiles over “long” genes (n = 2,875).

### Data and Code Availability

The accession number for all sequencing data reported in this study is GEO: GSE132400. The raw data used in this study have been deposited to Mendeley data and are available at https://doi.org/10.17632/8hzcg3bk37.1 .

## Supplemental Information

Document S1. Figures S1–S5 and Table S4

Table S1. Label-free Protein Quantification of FLAG-TFIIS_mut_ and RNAPII Immunoprecipitates, Related to Figure 1

Table S2. Differentially Expressed Genes in TFIIS_mut_ Cells, Related to Figures 3 and S3

Table S3. MISO Splicing Event, Related to Figure 3

Document S2. Article plus Supplemental Information
