# Supplementary material for: Elongation Factor TFIIS Prevents Transcription Stress and R-Loop Accumulation to Maintain Genome Stability
Source: Mol Cell. 2019 Oct 3;76(1):57–69.e9. doi: 10.1016/j.molcel.2019.07.037 (PMC6863433; doi:10.1016/j.molcel.2019.07.037)
Supplement: Document S1. Figures S1–S5 and Table S4 [file mmc1.pdf]

**Molecular Cell, Volume *76***

## Supplemental Information

# Elongation Factor TFIIS Prevents Transcription Stress and R-Loop Accumulation to Maintain Genome Stability

**Diana Zatreanu, Zhong Han, Richard Mitter, Emanuela Tumini, Hannah Williams, Lea Gregersen, A. Barbara Dirac-Svejstrup, Stefania Roma, Aengus Stewart, Andres Aguilera, and Jesper Q. Svejstrup**

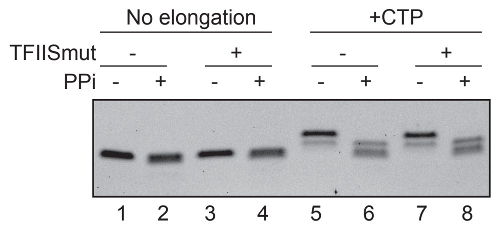


**Figure S1 (related to Figure 1). Little or no effect of TFIIS$_{mut}$ on RNAPII transcript cleavage induced by pyrophosphate.** RNAPII was transcribed two nucleotides, nucleotides removed, and TFIIS$_{mut}$ (1 μM) and pyrophosphate (PPi; 5mM) added as indicated.

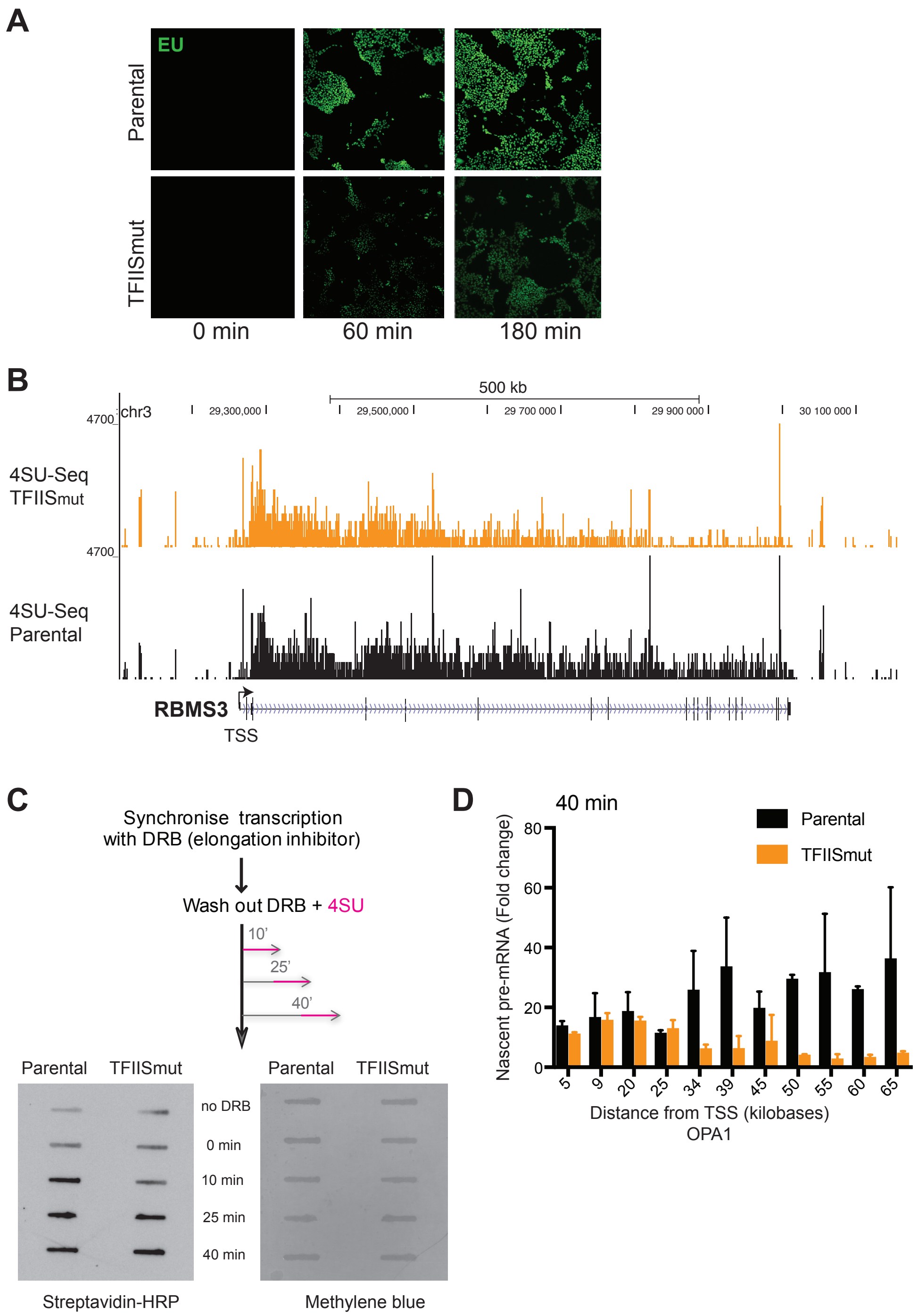


**Figure S2 (related to Figure 2). Decreased Nascent transcription and slow elongation in TFIIS$_{mut}$-expressing cells. A.** Nascent EU-labelled RNA shown in green at different time points of EU incorporation, 10x objective image. Representative images of TFIIS$_{mut}$ and parental cells. **B.** Representative example of the TT-seq profile, across the *RBMS3* gene. Notice the relative accumulation of 5'-end reads in the TFIIS$_{mut}$-expressing cells. See another example in Figure 2C. **C. Upper,** Experimental design schematics. **Lower,** Representative slot blot analysis of nascent RNA. After 3hr incubation with DRB, followed by wash out, cells were labelled for 10 min with 4SU, and different time points were harvested. Streptavidin was used to detect incorporated 4SU. Methylene blue staining is shown as loading control. **D.** Nascent RNA production 40 min after DRB release measured along the *OPA1* gene. *N=3,* Means ±SEM. (bars) are shown.

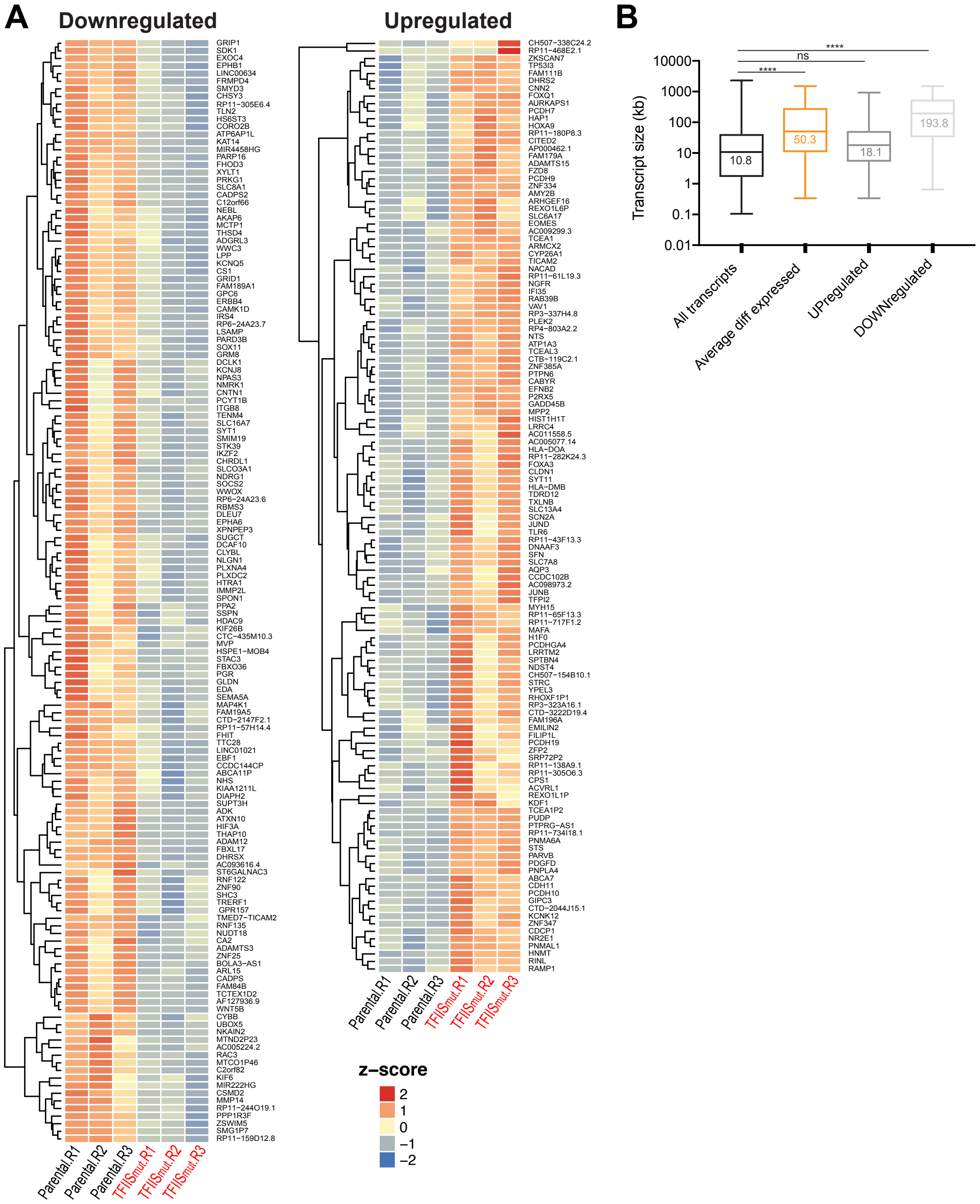


**Figure S3 (related to Figure 3). Differentially expressed genes after transcript cleavage inhibition. A.** Hierarchical clustering of downregulated (left) and upregulated (right) genes in $TFIIS_{mut}$ expressing cells, relative to expression in parental cells, *N*=3 biological replicates. Signals are scaled to raw *z*-scores of the rows. **B.** Downregulated genes in the $TFIIS_{mut}$-expressing cells are generally long compared with all induced genes. The whiskers denote minimum to maximum. *P*-values were determined by *t*-test.

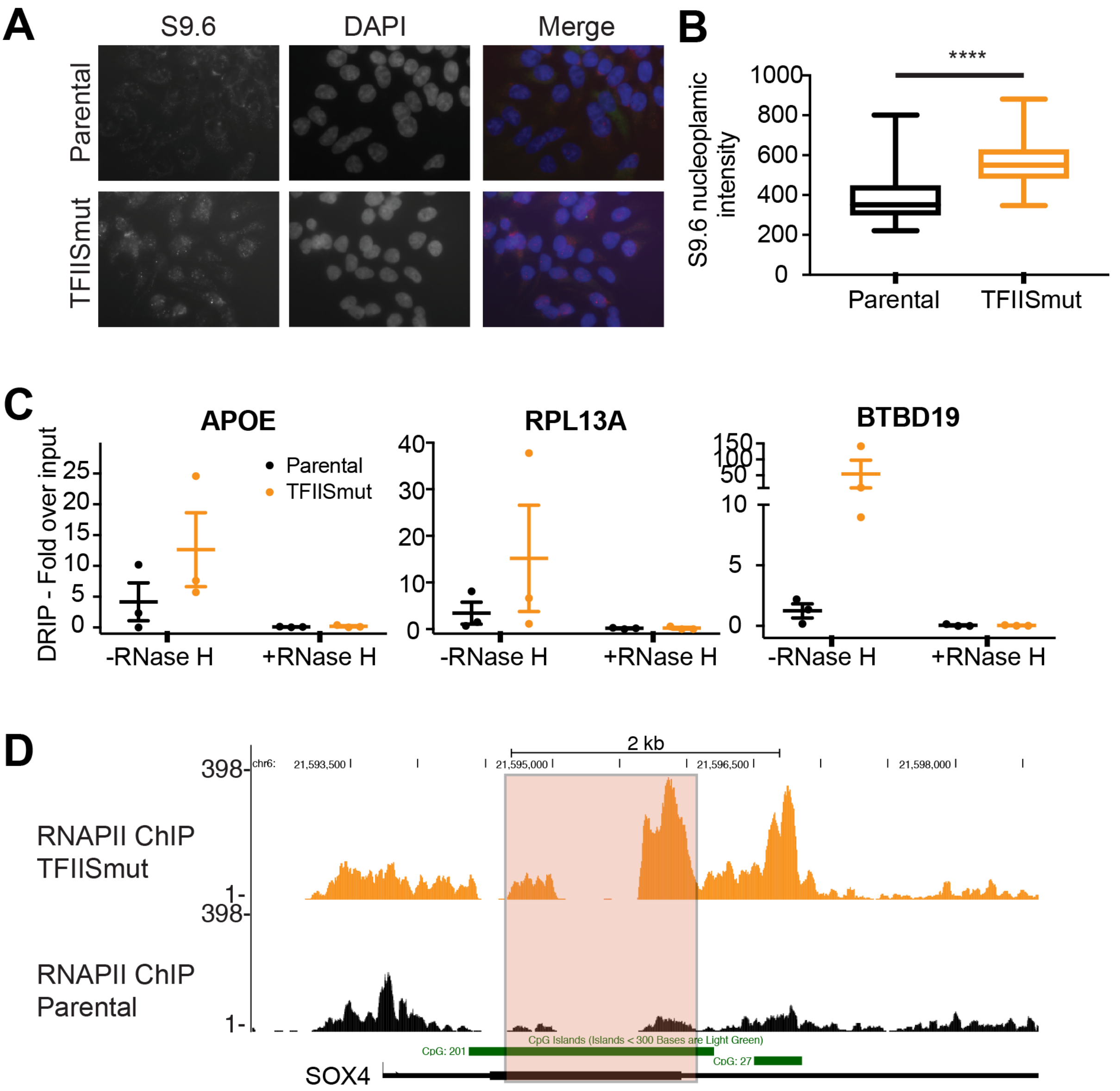


**Figure S4 (related to Figure 4). R-loops in TFIIS$_{mut}$-expressing cells. A.** Representative images of S9.6 immunostaining. **B.** Quantification of nuclear S9.6 intensity *N*=3. Means ±SEM. (bars) are shown. *P*-values were determined by two-way ANOVE t-test. **C.** DRIP-qPCR analysis of R-loop induction on the *APOE, RPL13A, BTBD19* genes; *N*=3. Means ±SEM. (bars) are shown. **D.** Areas in the *SOX4* gene with high GC-content (green bar), and with high RNAPII density in cells expressing TFIIS$_{mut}$ (orange peaks). The overlapping area tested by DRIP-qPCR is indicated by box.

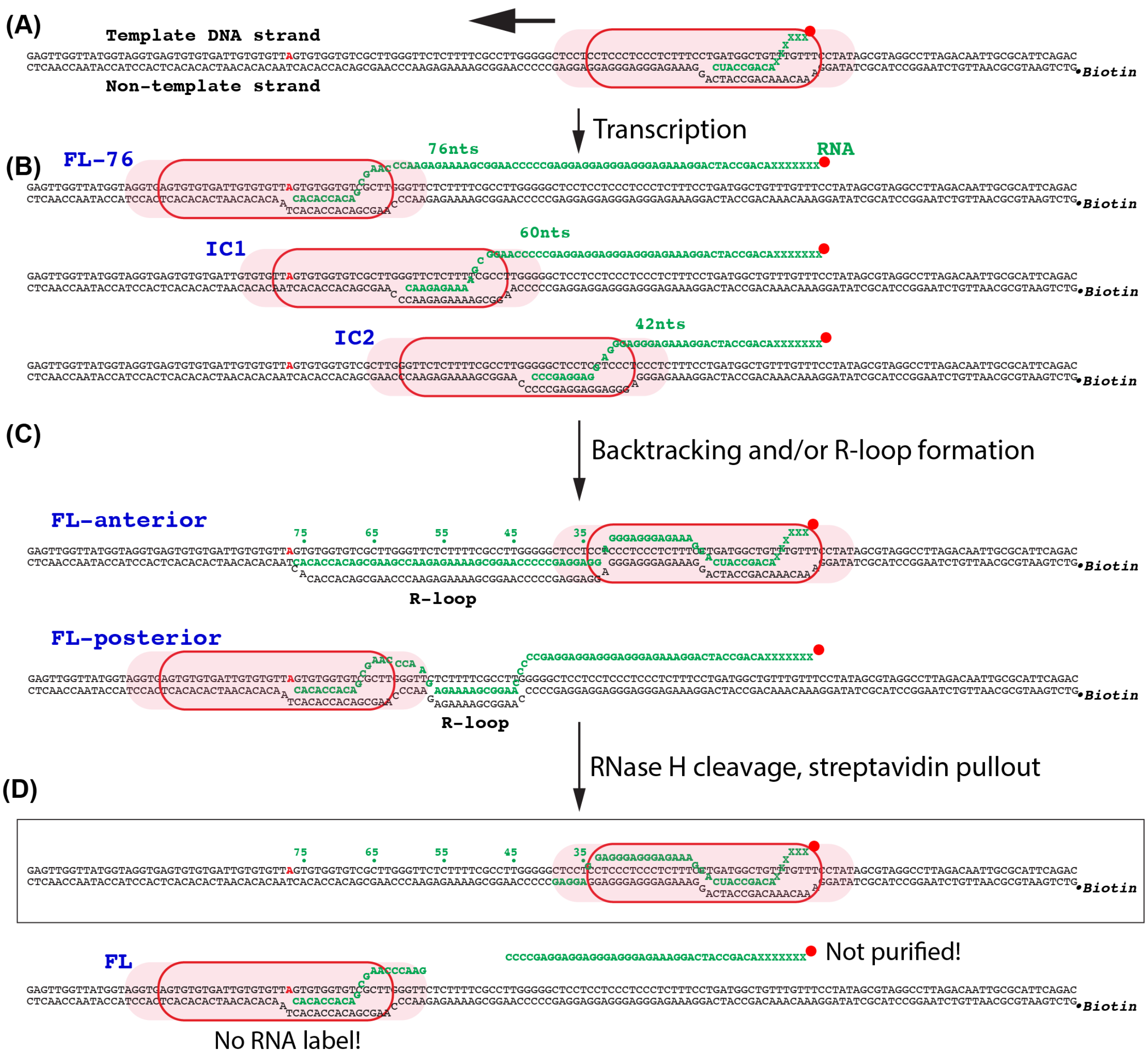


**Figure S5 (related to Figure 4). Evidence for TFIIS$_{mut}$-stimulated anterior R-loops in vitro. (A)** Initial TEC. Area protected by RNAPII (DNase I footprinting, area in light red; crystallography models, red line) and the transcription bubble, drawn to scale. Note that these features of elongating RNAPII are largely maintained during both forward and retrograde translocation (see (Saeki and Svejstrup, 2009) for details). Red dot indicates radioactive (or FAM-) label. The biotin-tag on the scaffold is indicated as well. Large arrow indicates direction of transcription (right to left). **(B)** Transcription products: full length (FL) and examples of intermediate transcription products (IC1 and IC2). The latter only occurred in the experiments of Figure 4F. **(C)** Products resulting from induced backtracking and/or R-loop formation. For simplicity, only R-loops from RNAPIIs generating FL transcripts are shown, but the fate of both anterior and posterior R-loops generated by IC1- and IC2-polymerases in (D) would be similar. Note that RNAPII will backtrack no further than to the start-point due to the unhybridizable nucleotides near the label; only this, fully backtracked RNAPII is shown. **(D)** Products of RNase H digestion. RNase H-mediated cleavage of R-loops results in RNAs of 30-40 nts in Figures 4E and F. This fits with the expectations from the 'footprint' of RNAPII (boxed example). Note that for any *posterior* R-loops (lower example), the label on RNA will lose connection to the immobilized template upon biotin purification, so these would not be detected by denaturing PAGE in Figure 4F.
